# Application of Voronoi Polyhedra for Analysis of Electronic Dimensionality in Emissive Halide Materials

**DOI:** 10.1021/jacs.4c14554

**Published:** 2024-12-10

**Authors:** Sergei A. Novikov, Hope A. Long, Aleksandra D. Valueva, Vladislav V. Klepov

**Affiliations:** Department of Chemistry, University of Georgia, Athens, Georgia 30602, United States

## Abstract

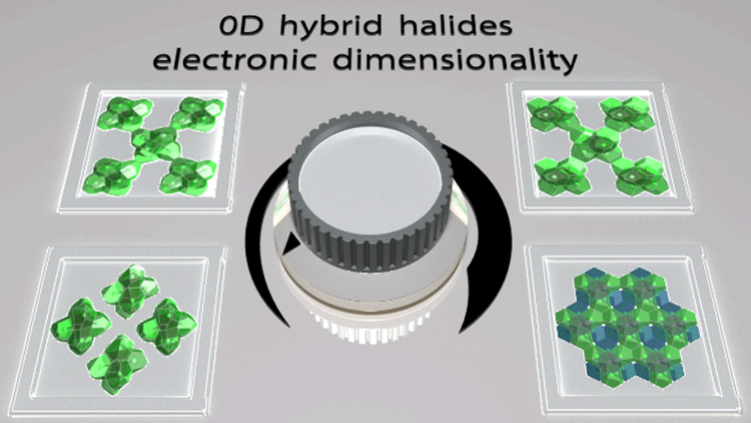

The synthesis of new hybrid halide materials is attracting increasing research interest due to their potential optoelectronic applications. However, general design principles that explain and predict their properties are still limited. In this work, we attempted to reveal the role of intermolecular interactions on the optical properties in a series of hybrid halides with an (Et_*n*_NH_4–*n*_)_2_Sn_1–*x*_Te_*x*_Cl_6_ (*n* = 1–4) composition. DFT calculations showed that the dispersions of the bands involving the Te 5s orbital character gradually decrease as the size of the organic cation increases, indicating a reducing orbital overlap between neighboring TeCl_6_^2–^ complexes. We characterized the photoluminescence (PL) of the Sn/Te solid solutions in (Et_*n*_NH_4–*n*_)_2_Sn_1–*x*_Te_*x*_Cl_6_ (*n* = 1–4) phases to correlate the electronic and optical properties. The PL response shows no concentration quenching effects in the (Et_4_N)_2_Sn_1–*x*_Te_*x*_Cl_6_ series, which demonstrated electronically isolated TeCl_6_^2–^ complexes. However, the series with smaller organic cations (*n* = 1–3) and higher electronic dimensionality show concentration quenching effects, which decrease as a function of the Te 5s band dispersions in these compounds. Similar trends can be revealed using a simple semiquantitative electronic dimensionality analysis method by means of Voronoi polyhedra. Since this approach relies only on structural data, it enables rapid characterization of orbital overlap between metal halide complexes in hybrid materials without DFT calculations. The present results allow us to conclude that electronic dimensionality plays an essential role in the photophysical properties of hybrid halide compounds and can be used to fine-tune their properties.

## Introduction

Compositional tunability and almost limitless structural variations in hybrid organic–inorganic metal halides (OIMHs) have made them one of the most widely studied classes of contemporary materials. Apart from fundamental interest in OIMHs, their remarkable optoelectronic properties, tunable band gaps, broadband photoluminescence, and high photoluminescence quantum yields make them excellent candidates for solar cell,^1−8^ light-emitting diode,^9−12^ radiation detector, and photodetector applications.^13−21^ While hybrid lead halide semiconductors with perovskite and perovskite-related structures remain the benchmark for photovoltaics, other classes of hybrid materials have been studied to circumvent the use of toxic lead and expand the new materials library.^22−28^ Particular interest has been paid to cations with an *n*s^2^ electron configuration and transition metal ions for the synthesis of air-stable lead-free OIMHs.^29−37^

The tremendous structural diversity of halides with *n*s^2^ ions is contributed by various coordination modes of metal cations, different dimensionalities of the structures, and a vast choice of organic cations. Besides the most common MX_6_ octahedral coordination (M = main group elements, X = halides), cations with stereoactive *n*s^2^ lone pairs can form MX_3_ pyramids, MX_4_ disphenoids, or MX_5_ square pyramids.^38^ MX_*n*_ units’ connection defines the structure’s dimensionality: corner-, edge-, and rarely face-sharing MX_*n*_ polyhedra form 3D frameworks, 2D layers, 1D chains, or 0D mono- and polynuclear anionic units. To balance the negative charge of metal halide units, various organic amines, which can act as structure-directing agents,^39,40^ are employed. The dimensionality of the OIMHs defines their properties and practical applications. Extended structure semiconductors favor properties, such as high charge carrier mobility, that are required for photovoltaics and photodetection.^41−43^ On the other hand, the extended structures demonstrate lower luminescence quantum yields and are more prone to photon reabsorption due to lower Stokes shifts of their luminescence compared to 1D and 0D materials.^32,34,44^ On the contrary, typically broad emission of 1D and 0D materials makes them perfect for luminescence applications.

One general assumption commonly made when studying 0D hybrid compounds is the isolated nature of the metal centers in these materials. Since 0D hybrid halides show no direct M–X–M bonds, this assumption is partially justified. However, the importance of anion interactions in some purely inorganic 0D halide compounds has been realized to contribute to band dispersions, increasing the charge carrier mobilities and enabling the resonant transfer of excitons in these compounds.^45^ In some cases, their conductivity is on par with materials that have generally recognized semiconducting properties due to their framework metal halide structures.^46^ For example, halide interactions are essential in Cs_2_SnI_6_ and Cs_2_TeI_6_, which have vacancy-ordered double perovskite structures.^47^ From a structural chemistry point of view, both of these compounds are built from isolated [SnI_6_]^2–^/[TeI_6_]^2–^ octahedra and Cs^+^ cations, representing a typical example of 0D structure. However, their electronic structures consist of highly dispersive bands due to interactions between the halogen atoms, rendering them with high charge carrier mobilities. While the importance of halogen orbital interactions has been realized, their influence on the properties of hybrid materials remains underexplored.^48−52^ Moreover, the elucidation of the electronic structures of these compounds requires somewhat costly DFT calculations, especially when it comes to hybrid functionals, which generally are not readily available to chemists. Because the electronic coupling in compounds depends on their crystal structures, information about interactions between the metal halide complexes should be available, at least in some approximation, from crystallographic data. However, no robust and universal tool for such an assessment has been developed so far.

**Figure 1:**
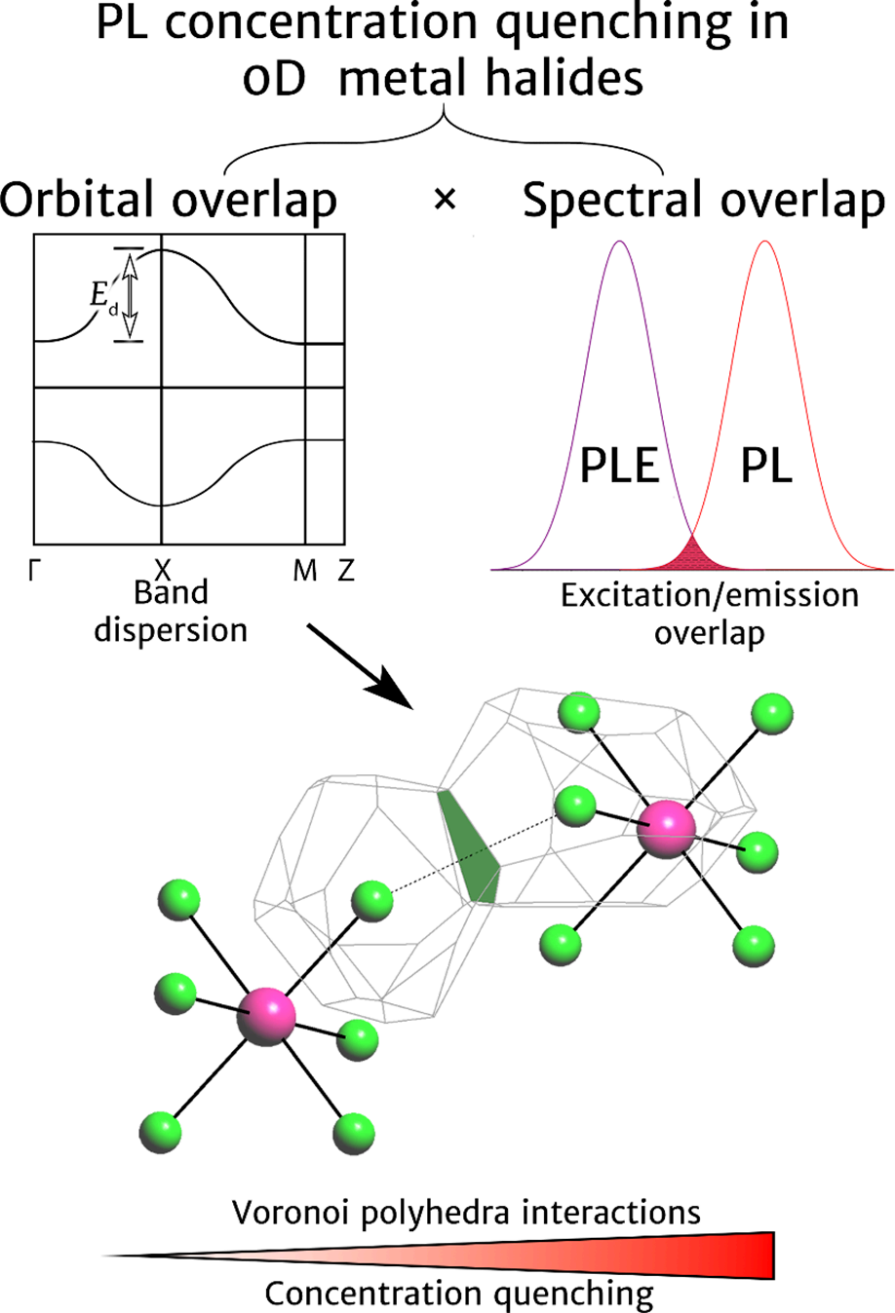
Schematic representation of two major components of energy transfer and photoluminescence quenching in 0D organic–inorganic metal halides.

In this report, we aim to develop tools that enable a simple assessment of halogen orbital overlap in hybrid halide materials. As a platform system, a series of tin and tellurium chloride compounds with an A_2_MCl_6_ general composition (A = (C_2_H_5_)_*n*_NH_4–*n*_^+^, *n* = 1–4, M = Sn or Te) has been selected. This system allows for convenient variation of the interactions between the MCl_6_ octahedra as a function of the organic cation volume (Figure 1). DFT calculations showed a changing degree of band dispersions in the series, from disperse to flat bands when *n* changes from 1 to 4. The photoluminescence properties of solid solutions with isomorphous Sn substitution for Te show agreement with the DFT data: PL concertation quenching effects are reduced as *n* increases. Importantly, we showed that the orbital overlap between the metal halide complexes could not be considered as a function of the metal–metal distance in the structure, and the mutual orientation of the complexes must be taken into account. Finally, the implementation of the Voronoi tessellation method for the analysis of intermolecular interactions between MCl_6_ complexes showed very promising results by providing semiquantitative data without time-consuming DFT calculations. We expect that further utilization of this method will enable a rational structural description to design highly efficient optoelectronic hybrid materials.

## Results and Discussion

### Crystal Structures

We used single-crystal X-ray diffraction to characterize four new compounds with the A_2_TeCl_6_ general composition (A = Et_*n*_NH_4–*n*_^+^, Et = −C_2_H_5_, *n* = 1–4, Tables S1–S22). (EtNH_3_)_2_TeCl_6_ crystallizes with the trigonal space group *P*3̅*m*1 with single crystallographically unique Te and Cl atoms occupying 1*b* and 6*i* Wyckoff positions. The ethylammonium cations are disordered over a 3-fold rotation axis. Both (Et_2_NH_2_)_2_TeCl_6_ and the monoclinic polymorph of (Et_3_NH)_2_TeCl_6_ (m-(Et_3_NH)_2_TeCl_6_) crystallize with the same *P*2_1_/*n* space group and have similar unit cell parameters and structural unit arrangements (Table S1). A single Te atom occupies the 2*a* Wyckoff position in both structures, while three independent Cl atoms are in general 4*e* positions (Table S2). The orthorhombic polymorph of (Et_3_NH)_2_TeCl_6_ (o-(Et_3_NH)_2_TeCl_6_) crystallizes with the *Pbca* space group. Unlike the other three hybrid halide structures discussed here, this phase has two independent Te atoms in the general 8*c* Wyckoff position.

**Figure 2:**
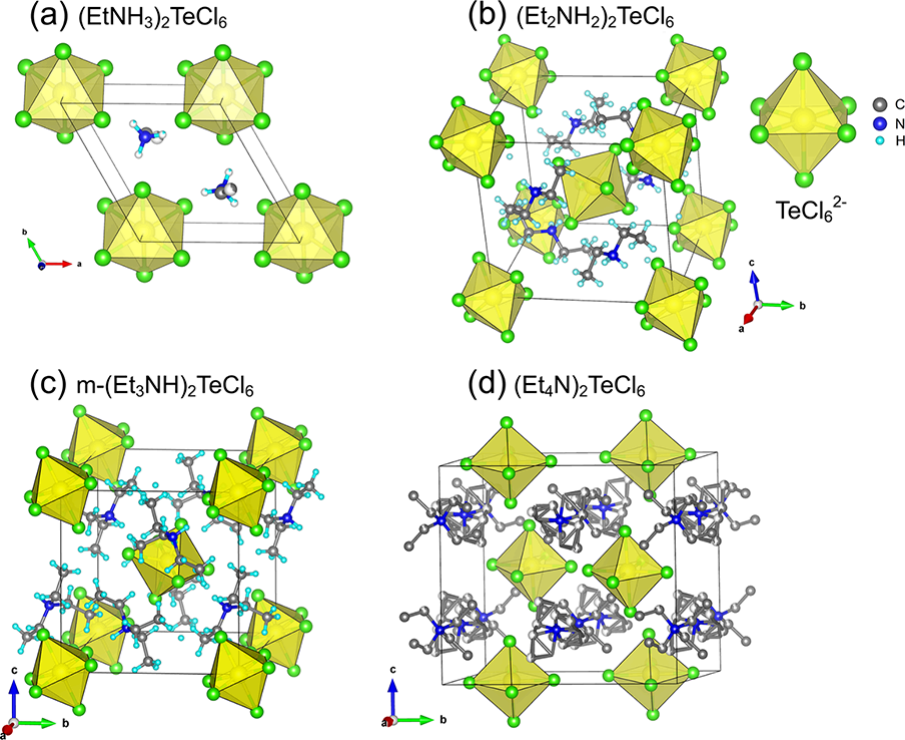
Views on the structures of hybrid tellurium halides of interest: (a) (EtNH_3_)_2_TeCl_6_, (b) (Et_2_NH_2_)_2_TeCl_6_, (c) m-(Et_3_NH)_2_TeCl_6_, and (d) (Et_4_N)_2_TeCl_6_.^53^ The yellow octahedra are TeCl_6_^2–^, Cl atoms are green, C atoms are gray, N atoms are blue, and H atoms (if located) are light blue.

In all new structures with an A_2_TeCl_6_ composition, each Te^4+^ cation is coordinated by six Cl^–^ anions in an octahedral manner. The octahedral TeCl_6_^2–^ units are isolated from each other, and their negative charges are balanced by the organic cations (Figure 2). As expected, a hydrogen bonding system is present in the new compounds (Table S23). In the (Et_2_NH_2_)_2_TeCl_6_ structure, each chlorine in a TeCl_6_^2–^ octahedron participates in hydrogen bonding with diethylammonium cations. As a result, this structure has infinite layers formed by N–H···Cl bonds running parallel to the (101̅) plane (Figure S1). In m- and o-(Et_3_NH)_2_TeCl_6_, hydrogen bonding forms 0D units. In the m phase, the sole TeCl_6_^2–^ octahedron is connected to two Et_3_NH^+^ cations, while the o phase shows a distinct hydrogen bonding pattern: only one of two independent TeCl_6_^2–^ anions forms hydrogen bonds with four Et_3_NH^+^ cations (Figure S1).

### Electronic Structures

Recent detailed studies on Cs_4_PbBr_6_ and Cs_2_SnI_6_ showed that band structure dispersion is a convenient tool to assess noncovalent interactions between neighboring halide complexes.^45−47^ Although this approach is somewhat universal,^54^ it is rarely discussed in the context of hybrid halide materials, which usually show a greatly reduced degree of orbital overlap compared to 3D halide perovskites. While it is commonly accepted that the dimensionality of hybrid halides with structurally isolated AX_6_ octahedra is 0D, their electronic dimensionality is strongly affected by the mutual arrangement and interactions between the octahedra. This section begins with a discussion of the molecular orbital (MO) diagrams of the SnCl_6_ and TeCl_6_ octahedral complexes. After introducing the MOs, we consider their interactions in a structure by paying close attention to the dispersion of bands formed by the Sn 5s, Te 5s, and Te 5p orbitals.

**Figure 3:**
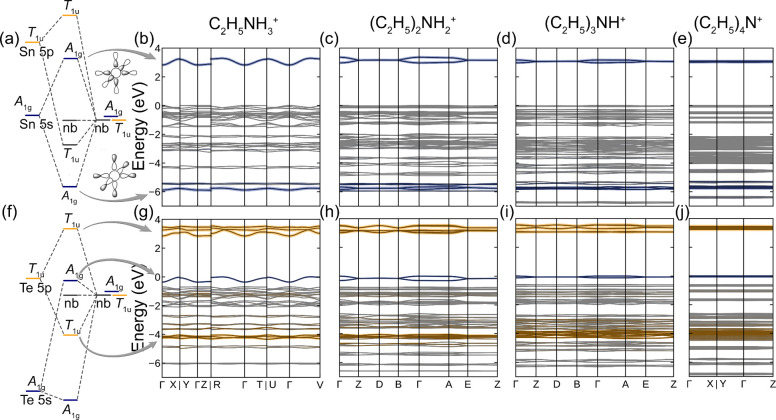
(a,f) Schematic molecular orbital diagrams of octahedral SnCl_6_ and TeCl_6_ complexes. (b–e) Band structure diagrams of ((C_2_H_5_)_*n*_NH_4*–n*_)_2_SnCl_6_ and (g–j) ((C_2_H_5_)_*n*_NH_4*–n*_)_2_TeCl_6_ (*n* = 1–4). A_1g_ MO orbitals (σ bonds formed by Sn/Te 5s and Cl 3p orbitals) are highlighted with blue, and T_1u_ orbitals (σ and π bonds formed by Te 5p and Cl 3p orbitals) are highlighted with orange.

Schematic molecular orbital diagrams of SnCl_6_ and TeCl_6_ complexes are given in Figure 3.^46^ In the SnCl_6_ MO diagram, the Sn 5s and Cl 3p orbitals with A_1g_ symmetry form a pair of bonding and antibonding orbitals. The interaction of the T_1u_ Sn 5p and Cl 3p orbitals results in another pair of bonding and antibonding orbitals at different energy levels. The remaining Cl 3p orbitals form numerous nonbonding states. For Sn^4+^, both the antibonding A_1g_ and T_1u_ orbitals hold no electrons. Thus, lower-energy A_1g_ orbitals form the conduction band in the Sn^4+^X_6_-containing periodic compounds. In contrast, the valence bands are contributed by nonbonding Cl 3p orbitals, making a transition from the HOMO to LUMO (from the valence band to the conduction band in a periodic compound) a nominal ligand-to-metal charge transfer transition. Unlike Sn^4+^, more electronegative Te^4+^ retains its 5s^2^ electrons in the TeCl_6_^2–^ complexes. Due to a large energy difference between Te 5s and Cl 3p orbitals, their overlap causes only a small energy splitting, resulting in antibonding A_1g_ orbitals being slightly higher in energy than nonbonding Cl 3p orbitals. Larger overlap between Te 5p and Cl 3p orbitals leads to greater energy splitting of the resulting bonding and antibonding MOs. Due to this, the LUMO orbitals are contributed by Te 5p and Cl 3p T_1u_ orbitals. Since both the HOMO and LUMO of the Te^4+^Cl_6_ complexes are contributed by Te orbitals, 5s for the HOMO and 5p for the LUMO, one can consider an electron excitation as a well-known 5s^2^5p^0^ → 5s^1^5p^1^ transition, assuming that there are no orbital interactions between neighboring TeCl_6_ complexes. In periodic compounds with isolated TeCl_6_^2–^ complexes, MOs mostly retain their individuality, often resulting in flat or nearly flat bands.

Although 0D hybrid structures contain structurally distinct octahedral units with no apparent covalent bonds between them, noncovalent interactions can contribute to the corresponding band dispersion.^45^ To study this effect in greater detail, we calculated the band structures of all eight compounds in the A_2_MCl_6_ (A = Et_*n*_NH_4*–n*_^+^*,* Et = −C_2_H_5_, *n* = 1–4, M = Sn or Te) family (Figure 3). In the case of the bulky cation Et_4_N^+^, the band structures consist of almost perfectly flat bands corresponding to the isolated MOs of the SnCl_6_ or TeCl_6_ complexes. The lack of band dispersions indicates nearly no orbital interactions between the complexes, which are structurally and electronically isolated by organic cations, thus making (Et_4_N)_2_SnCl_6_ and (Et_4_N)_2_TeCl_6_ truly 0D compounds. As the size of the organic cation decreases, the band dispersion increases. Since the A_1g_ MO in MCl_6_ complexes consists of Cl 3p orbitals that form σ bonds and extend further away from the complex (Figure 3a), we chose Sn 5s and Te 5s band dispersions to assess the interactions between the complexes (Figure S2). As expected, the most dispersed bands are observed in the (EtNH_3_)_2_MCl_6_ (M = Sn or Te) electronic structures, where the bands with Sn 5s and Te 5s characters have dispersions of 0.486 and 0.368 eV, respectively. These values indicate that the ethylammonium cations’ sizes are insufficient to shield the MCl_6_ octahedra completely. Increasing the cation size by one ethyl group results in lower band dispersions of 0.401 and 0.314 eV for (Et_2_NH_2_)_2_MCl_6_, where M = Sn and Te, respectively. Interestingly, in a very similar structure of (Et_3_NH)_2_MCl_6_, the band dispersions are twice as small as those in the diethyl counterpart, 0.202 and 0.127 for Sn and Te, respectively. Although the change in the band dispersion shows a gradual decrease in the orbital overlap between neighboring octahedra, it is not directly proportional to the M···M distance in these structures. This somewhat unexpected trend can be illustrated in the Sn series, where the shortest experimental Sn···Sn distances of 7.27, 8.40, 8.51, and 9.59 Å correspond to the dispersions of 0.486, 0.401, 0.202, and <0.001 eV. Although these values of dispersion are significantly less than the ranges that are typically observed for 3D perovskites (>2 eV)^55,56^ or vacancy-ordered double perovskite (∼1 eV),^47^ they can be sufficient to promote electronic coupling between the complexes. These energy interactions can be characterized by measuring the optical properties of the A_2_Sn_1–*x*_Te_*x*_Cl_6_ solid solutions.

### Photoluminescence Properties of ((C_2_H_5_)_*n*_NH_4–*n*_)_2_Sn_1–*x*_Te_*x*_Cl_6_ (*n* = 1–4) Solid Solutions

Since (Et_*n*_NH_4*–n*_)_2_TeCl_6_ phases are isostructural with (Et_*n*_NH_4*–n*_)_2_SnCl_6_ compounds with the same organic cations (Table S1),^53,57−61^ the isomorphous substitution of Sn^4+^ for Te^4+^ in all ranges of concentrations is highly favorable. The varying degree of band dispersions makes these solid solutions highly suitable for characterizing energy exchange as a function of Te dopant concentration. Due to this, we synthesized four series of solid solutions and characterized their photoluminescence properties.

**Figure 4:**
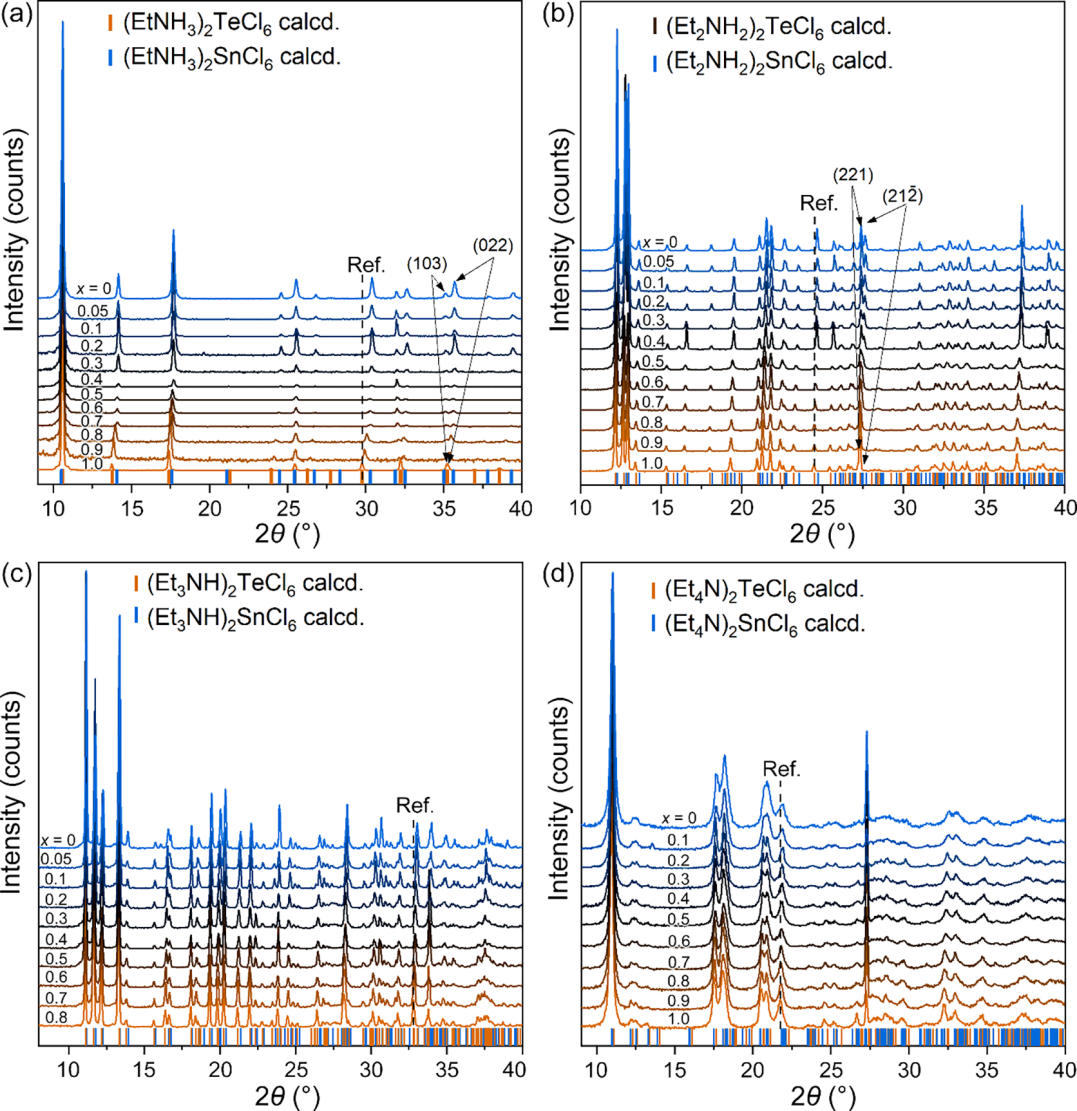
PXRD patterns of the (Et_*n*_NH_4*–n*_)_2_Sn_1–*x*_Te_*x*_Cl_6_ series: (a) (EtNH_3_)_2_Sn_1–*x*_Te_*x*_Cl_6_, (b) (Et_2_NH_2_)_2_Sn_1–*x*_Te_*x*_Cl_6_, (c) (Et_3_NH)_2_Sn_1–*x*_Te_*x*_Cl_6_, and (d) (Et_4_N)_2_Sn_1–*x*_Te_*x*_Cl_6_. Ref. stands for the reference line; (Et_4_N)_2_Sn_1–*x*_Te_*x*_Cl_6_ samples were collected with the Ge powder as an internal standard.

**Figure 5:**
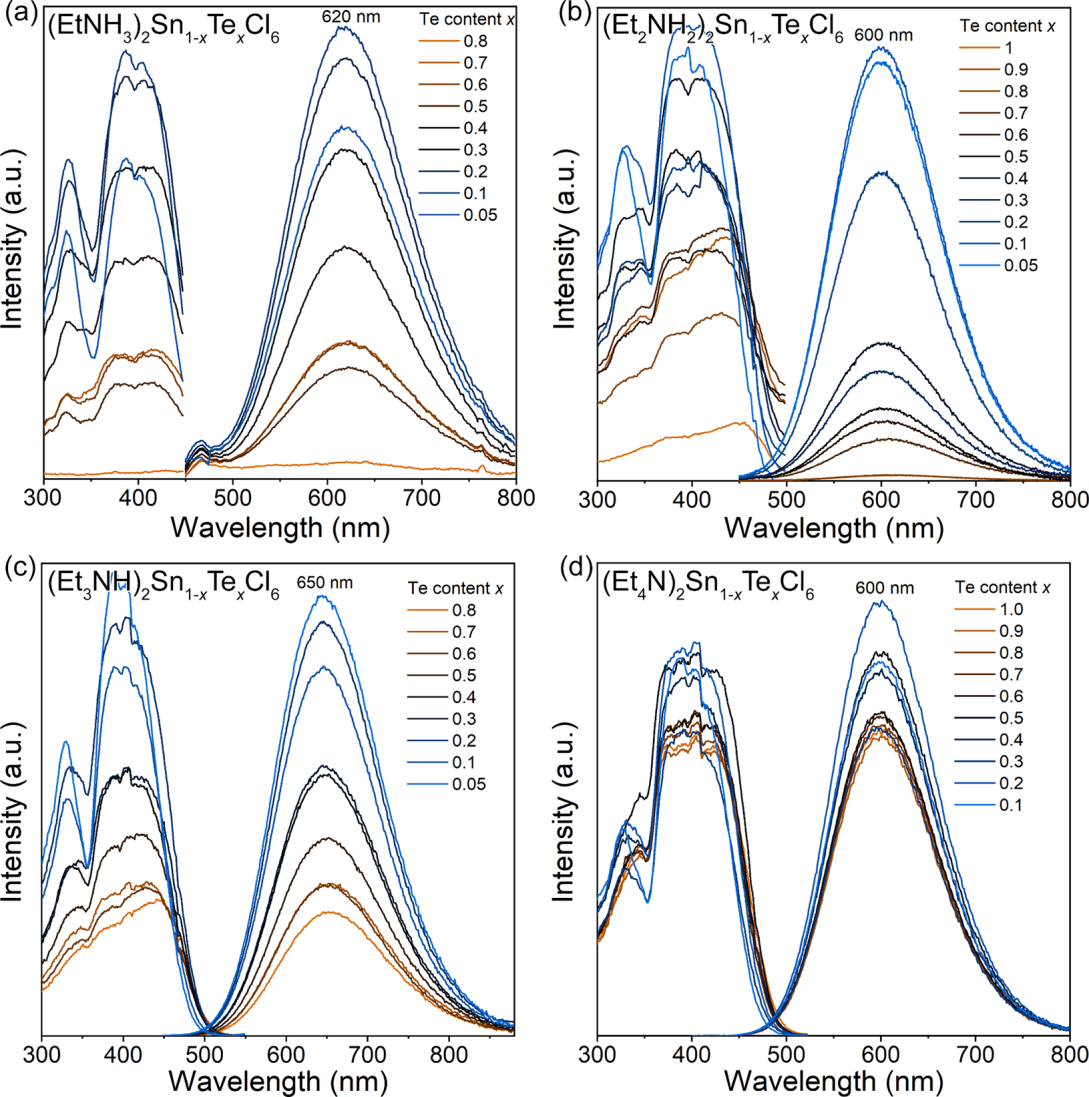
PLE and PL spectra of (Et_*n*_NH_4*–n*_)_2_Sn_1–*x*_Te_*x*_Cl_6_ series: (a) (EtNH_3_)_2_Sn_1–*x*_Te_*x*_Cl_6_ (*x* = 0.05–0.80, λ_ex._ = 388 nm, and λ_em._ = 620 nm), (b) (Et_2_NH_2_)_2_Sn_1–*x*_Te_*x*_Cl_6_ (*x* = 0.05–1.00, λ_ex._ = 375 nm, and λ_em._ = 600 nm), (c) (Et_3_NH)_2_Sn_1–*x*_Te_*x*_Cl_6_ (*x* = 0.05–0.80, λ_ex._ = 405 nm, and λ_em._ = 650 nm), and (d) (Et_4_N)_2_Sn_1–*x*_Te_*x*_Cl_6_ (*x* = 0.1–1.0, λ_ex._ = 395 nm, and λ_em._ = 600 nm).

In all four series of the hydrothermally prepared (Et_*n*_NH_4*–n*_)_2_Sn_1–*x*_Te_*x*_Cl_6_ solid solutions (*x* = 0.0–1.0), along with selected compounds from the (Et_2_NH_2_)_2_Sn_1–*x*_Te_*x*_Cl_6_ series prepared by solid-state reactions, gradual diffraction peak shifts were observed (Figure 4). Bragg peak splitting is another clear sign of successful solid solution formation. For example, in the pristine (EtNH_3_)_2_TeCl_6_ phase, two peaks corresponding to the (103) and (022) families of crystallographic planes are separated by ≈0.06° 2θ, while in (EtNH_3_)_2_SnCl_6_, this separation is ≈0.6°, which can be easily detected experimentally (Figure 4). Similarly, peak splitting is observed for the (Et_2_NH_2_)_2_MCl_6_ phases at around 27° (Figure 4). Additionally, we collected EDS data (Figure S3 and Table S24) to confirm Te incorporation in the desired ratios. Raman spectroscopy can also serve as evidence of the solid solution formation. Pristine phases feature two peaks associated with ν_1_ and ν_2_ bands of the MCl_6_^2–^ anions in the low-frequency region.^62−65^ In comparison, the Raman spectrum of an (Et_2_NH_2_)_2_Te_0.5_Sn_0.5_Cl_6_ sample with three peaks appears as an overlap of the pristine phase spectra (Figure S6).

(Et_*n*_NH_4*–n*_)_2_Sn_1–*x*_Te_*x*_Cl_6_ samples emit visible light in the orange-red region under UV-light excitation. The corresponding photoluminescence excitation (PLE) and emission (PL) data are listed in Figure 5. Independent of the organic cation, the photoluminescence of all (Et_*n*_NH_4–*n*_)_2_Sn_1–*x*_Te_*x*_Cl_6_ samples features a broad asymmetric excitation band and broad emission with one near-Gaussian shape peak. At lower concentrations of Te^4+^, the PLE spectra have two distinguished regions with maxima at ≈330 and ≈400 nm, while at high Te^4+^ concentrations, the regions overlap (Figure 5). These two regions are referred to as the A and B bands of *n*s^2^ ion in octahedral surroundings.^66,67^ The lower-energy A band originates in the spin-forbidden ^1^S_0_ → ^3^P_1_ transition. The A band is a doublet since the ^3^P_1_ level is split in the octahedral field by the dynamic Jahn–Teller effect, so the experimentally observed peak is asymmetric.^66^ The singlet B band originates in the forbidden electric dipole ^1^S_0_ → ^3^P_2_ transition, while the high-intensity and highest-energy C band arises from the spin-allowed ^1^S_0_ → ^1^P_1_ transition and usually lies in the UV-B region.^67^ PL spectra of (Et_*n*_NH_4*–n*_)_2_Sn_1–*x*_Te_*x*_Cl_6_ samples feature single broad (fwhm ≈ 110–130 nm, Figure S4) symmetric peaks.

The peak positions do not change significantly with *x* in (Et_*n*_NH_4*–n*_)_2_Sn_1–*x*_Te_*x*_Cl_6_ but depend on the organic cation. The lowest emission wavelengths (600 nm) are observed in the (Et_2_NH_2_)_2_Sn_1–*x*_Te_*x*_Cl_6_ and (Et_4_N)_2_Sn_1–*x*_Te_*x*_Cl_6_ series. (EtNH_3_)_2_Sn_1–*x*_Te_*x*_Cl_6_ samples have an emission peak at 620 nm. Finally, the emission of (Et_3_NH)_2_Sn_1–*x*_Te_*x*_Cl_6_ samples is redshifted to 650 nm. PL peaks correspond to the ^3^P_1_ → ^1^S_0_ relaxation of the Te^4+^ species.^68^ All four compounds have similar Stokes shifts of 1.20, 1.24, 1.15, and 1.07 eV for *n* = 1–4, respectively, indicating a similar spectral overlap among them.

The compounds with smaller organic cations, *n* = 1–3, show different PL dependence on the Te concentration from the *n* = 4 series. In the (Et_4_N)_2_Sn_1–*x*_Te_*x*_Cl_6_ series, the Te concentration has little effect on the PL intensity, indicating low energy exchange between the Te centers. This observation agrees well with the 0D electronic band structure of the (Et_4_N)_2_SnCl_6_ and (Et_4_N)_2_TeCl_6_ compounds, as evidenced by their nearly flat bands. Reducing the organic cation size in the compounds with *n* = 1–3 “switches on” the orbital overlap and energy exchange between the complexes. In all three series, the PL intensity increases as the concentration of Te decreases, showing prominent concentration quenching effects. The effect is especially remarkable in the (EtNH_3_)_2_Sn_1–*x*_Te_*x*_Cl_6_ series where samples with *x* ≥ 0.8 appear nonluminous, and Te dilution causes a multifold emission intensity increase. This observation is in excellent agreement with the theoretically calculated highest band dispersion in both (EtNH_3_)_2_MCl_6_ (M = Sn or Te) compounds.

**Figure 6:**
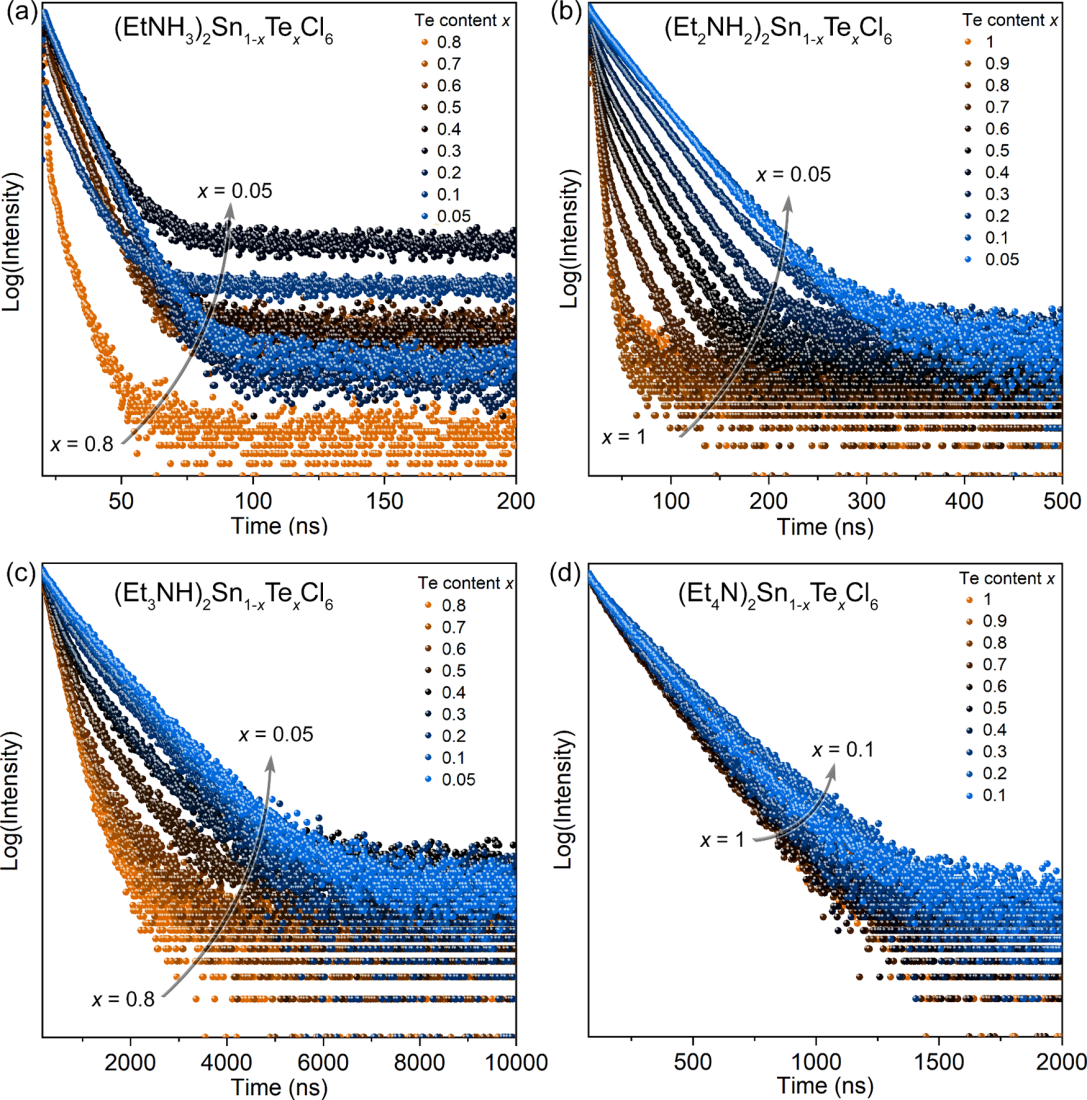
Time-resolved PL spectra of (Et_*n*_NH_4*–n*_)_2_Sn_1–*x*_Te_*x*_Cl_6_ series, λ_ex._ = 372 nm: (a) (EtNH_3_)_2_Sn_1–*x*_Te_*x*_Cl_6_ (*x* = 0.05–0.80), (b) (Et_2_NH_2_)_2_Sn_1–*x*_Te_*x*_Cl_6_ (*x* = 0.05–1.00), (c) (Et_3_NH)_2_Sn_1–*x*_Te_*x*_Cl_6_ (*x* = 0.05–0.80), and (d) (Et_4_N)_2_Sn_1–*x*_Te_*x*_Cl_6_ (*x* = 0.1–1.0).

The concentration effects on the PL lifetimes in the series from *n* = 1 to 4 provide additional experimental evidence for energy exchange reduction (Figure 6 and Table S25). Nearly absent orbital overlap between the octahedral complexes in the (Et_4_N)_2_Sn_1–*x*_Te_*x*_Cl_6_ series makes the PL lifetime independent of the Te concentration changes. The small EtNH_3_^+^ cation promotes the energy exchange that leads to a strong concentration quenching effect and shorter PL lifetimes at higher Te concentrations. Despite very similar structures and similar shortest Te···Te distances, the two intermediate series (Et_2_NH_2_)_2_Sn_1–*x*_Te_*x*_Cl_6_ and (Et_3_NH)_2_Sn_1–*x*_Te_*x*_Cl_6_ behave differently. In (Et_2_NH_2_)_2_Sn_1–*x*_Te_*x*_Cl_6_, which has a relatively strong orbital overlap between the complexes, the PL lifetime rapidly decreases from 36(1) ns for *x* = 0.05 to 4.0(1) ns for *x* = 0.8. The (Et_3_NH)_2_Sn_1–*x*_Te_*x*_Cl_6_ series that has half of the band dispersion compared to the Et_2_NH_2_ analog demonstrates significantly longer PL lifetimes varying from 959(6) to 278(6) ns for *x* = 0.05 and 0.8, respectively.

**Figure 7:**
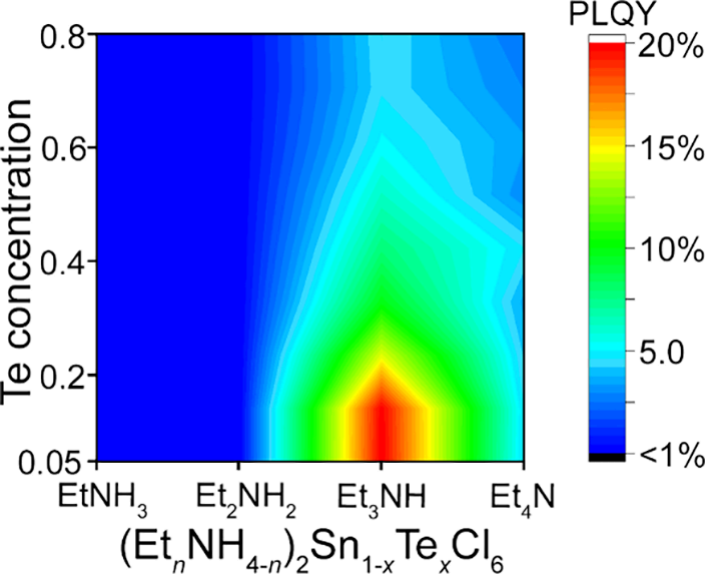
Photoluminescence quantum yield (PLQY) as a function of the organic cation and Te concentration.

Results of PLQY measurements for the (Et_*n*_NH_4*–n*_)_2_Sn_1–*x*_Te_*x*_Cl_6_ series also agree with the calculated energy exchange interactions (Figure 7). Samples with ethyl- and diethylammonium demonstrate low quantum yields (Table S26) below the measurement limit. For the (Et_3_NH)_2_Sn_1–*x*_Te_*x*_Cl_6_ series, PLQY values demonstrate significant growth with lowering Te^4+^ concentration, and the highest η = 20.5% was achieved in the (Et_3_NH)_2_Sn_0.95_Te_0.05_Cl_6_ sample. For the (Et_4_N)_2_Sn_1–*x*_Te_*x*_Cl_6_ series, no significant changes of PLQY were observed with the change of the Te^4+^ content: the experimental η values of all samples are close to those of the pristine (Et_4_N)_2_TeCl_6_ phase. Notably, the distortion levels of TeCl_6_^2–^ units cannot be considered as a factor predetermining higher lifetimes or quantum yields of the (Et_*n*_NH_4*–n*_)_2_Sn_1–*x*_Te_*x*_Cl_6_ phases. For example (Table S27), (EtNH_3_)_2_MCl_6_ (M = Sn or Te) has the lowest Δ_d_,^69^ while (Et_2_NH_2_)_2_SnTeCl_6_ the lowest Σ,^70^ yet both phases have PLQY near the background of the measurement.

### Assessment of Orbital Overlap between Halide Complexes in Hybrid Phases

Photoluminescence measurements show that the orbital overlap between the halide complexes plays a central role in the optical properties of these materials. While the lack of orbital overlap in (Et_4_N)_2_Sn_1–*x*_Te_*x*_Cl_6_ solid solutions results in independent TeCl_6_ centers that show no concentration quenching effects, their PLQY is also relatively low, likely because of the absence of energy exchange between the complexes. On the other hand, the presence of the strong orbital overlap in (EtNH_3_)_2_Sn_1–*x*_Te_*x*_Cl_6_ and (Et_2_NH_2_)_2_Sn_1–*x*_Te_*x*_Cl_6_ results in the dominant concentration quenching effects and, therefore, low PLQY of the solid solutions even at low Te concentrations. The optimal balance in orbital overlap and Stokes shift is achieved when Et_3_NH^+^ serves as an organic cation, which enables the highest among these series PLQY values in diluted (Et_3_NH)_2_Sn_1–*x*_Te_*x*_Cl_6_ solid solutions.

Despite the apparent importance of orbital overlap between the isolated octahedral complexes, its influence on the optical properties of hybrid materials remains largely underexplored, although some attempts have been made in recent years. For example, Mao et al. established the influence of interatomic Mn···Mn distances on PLQY of a series of hybrid manganese bromides.^71^ Similarly, Sb···Sb distances in hybrid antimony halides play an important role in defining PLQY of these compounds.^72^ These examples can be considered as one of the attempts to quantify interactions between halide complexes in optical hybrid materials, which in the case of the Mn hybrids showed that concentration quenching effects become predominant in compounds where Mn···Mn distances are below 9 Å. Although M···M distances seem to be an intuitive measure of interactions between the metal halide complexes, our attempt to implement it to the (Et_*n*_NH_4*–n*_)_2_Sn_1–*x*_Te_*x*_Cl_6_ (*n* = 1–4) series did not provide satisfactory results. For example, the shortest Sn···Sn distances in (Et_2_NH_2_)_2_SnCl_6_ and (Et_3_NH)_2_SnCl_6_, 8.40 and 8.51 Å, cannot explain a dramatic change in the optical properties of their solid solutions with Te. Moreover, these compounds have the same packing of cations and octahedral complexes in their structures, highlighting the importance of subtle changes in their structures for their optical properties.

**Figure 8:**
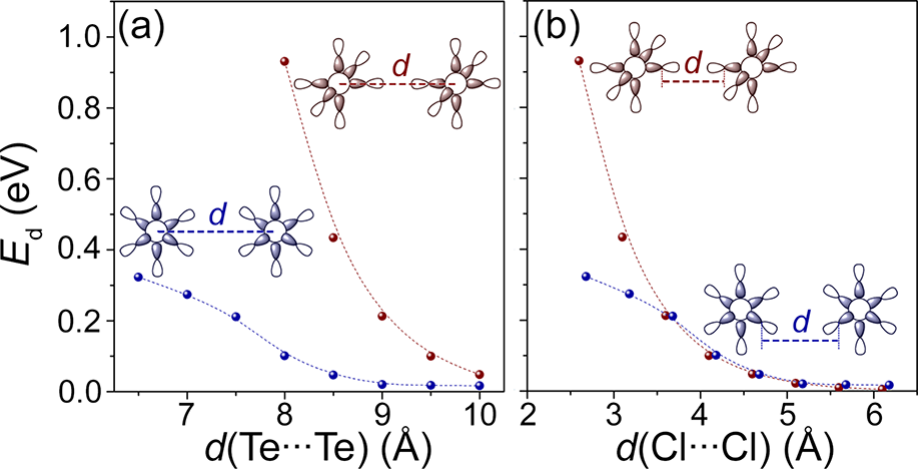
A_1g_ band dispersion energy as a function of (a) Te···Te or (b) Cl···Cl distances in chains of TeCl_6_ complexes. Two octahedral complex orientations are shown: linear (red plots) and rotated by 45° around the *z*-axis (blue plots).

To study the effect of mutual orientation of the MCl_6_ octahedra on exchange interactions, we performed a series of DFT calculations on chains of TeCl_6_ octahedra with two distinct orientations: in one case, the Cl–Te–Cl···Cl–Te–Cl fragments are linear (vertex to vertex); in the other one, they are rotated by 45° (edge to edge) as shown in Figure 8. The A_1g_ band dispersion plotted as a function of the interatomic Te distance indicates a strong dependence of the orbital overlap on the mutual orientation of the octahedra. As expected, the linear orientation results in much higher band dispersion even at significantly longer Te···Te distances. One apparent reason for this is shorter Cl···Cl distances for the linear configuration (Figure 8b). However, when the band dispersions are plotted as a function of Cl···Cl distances, the interactions become very similar when *d*(Cl···Cl) > 3.5 Å. The increasing difference in dispersions below 3.5 Å arises due to better orbital overlap in σ vs non-σ bonds and has little effect on noncovalent bonding. For example, the shortest Cl···Cl contact within a TeCl_6_ complex in (EtNH_3_)_2_TeCl_6_ is 3.50 Å, which can arbitrarily be set as the shortest noncovalent interaction between Cl atoms in real structures. Assuming this shortest bond limit, there is virtually no difference in band dispersions for these two mutual orientations. This allows one to come to a somewhat intuitive conclusion that the main deciding factor in orbital overlap between halide complexes is the shortest distance between halide atoms.

**Figure 9:**
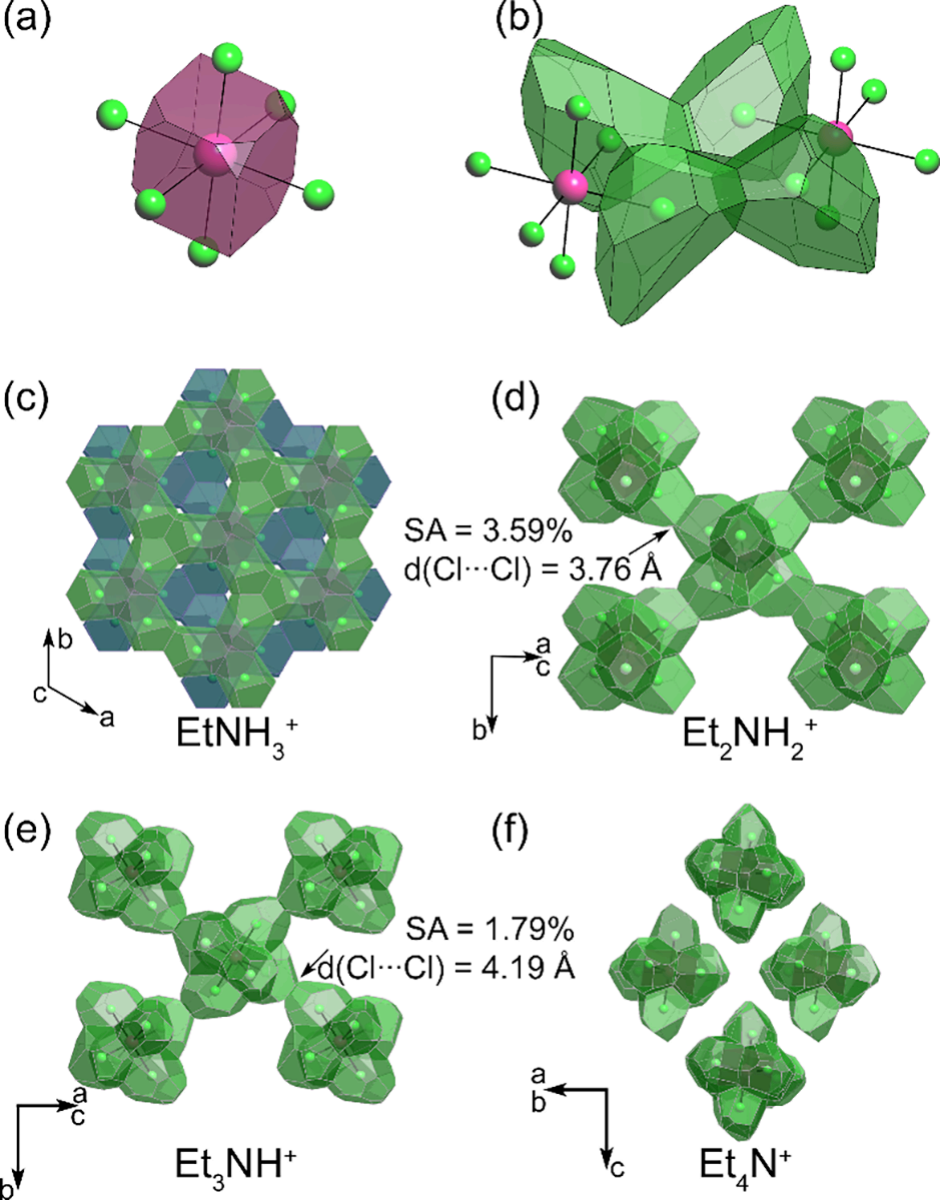
Voronoi polyhedra (VP) analysis of intermolecular interactions in (Et_*n*_NH_4*–n*_)_2_TeCl_6_ structures. (a) View on the Te atom VP in a [TeCl_6_]^2–^ complex. (b,c) Cl atom VPs show a strong interaction between two complexes. (d,e) Decreasing interaction between the octahedral TeCl_6_ complexes as *d*(Cl···Cl) and the corresponding solid angle decrease. (f) Electronically isolated TeCl_6_ complexes in (Et_4_N)_2_TeCl_6_.

While using simple geometric parameters such as Cl···Cl distances seems to be a straightforward way of assessing interactions between the complexes, this characterization can become tedious for all possible contacts in a structure. Voronoi polyhedra provide a more convenient visual and quantitative characterization of Cl···Cl interatomic contacts that also consider other structural fragments. A Voronoi polyhedron (VP) can be viewed as an atom’s electron density in a crystal structure, where each point of a Voronoi polyhedron is closer to its central atom than to any other atom in the structure (Figure 9a).^73^ One can, therefore, conclude the dimensionality and the strength of Cl orbital overlap by assessing the Cl atoms’ Voronoi polyhedra contacts. For example, a fragment of the (EtNH_3_)_2_TeCl_6_ structure is shown in Figure 9b. In this fragment, there are strong interactions between neighboring TeCl_6_ complexes, which correspond to short 3.77 Å contacts between the Cl atoms. These contacts correspond to relatively large solid angles, 4–5% of the total 2π steradian solid angle, of the Voronoi polyhedron (for comparison, valence bonds correspond to 5–21% solid angles).^73,74^ Strong Cl···Cl contacts form dense 2D slabs of overlapping Cl orbitals (Figure 9c). In each TeCl_6_ complex of the slabs, six Cl atoms form Cl···Cl contacts, corresponding to a total of 51.3% solid angle. These slabs have virtually no overlap since the shortest Cl···Cl contact between them is 5.9 Å long with a negligible total solid angle of 0.02%. This geometric analysis allows one to conclude that (EtNH_3_)_2_TeCl_6_ has a 2D electronic structure with strong orbital overlap between the TeCl_6_ complexes, which agrees with the DFT calculations (note the flat band in the Γ–*Z* direction vs disperse bands in the Γ–*X* and *Y*–Γ directions in Figure 3g).

Simple geometric structure analysis with Voronoi polyhedra also enables an understanding of decreasing orbital overlap interactions in the remaining series. The other end member of the series, (Et_4_N)_2_TeCl_6_, has no short Cl···Cl contacts in its structure. Therefore, the Cl Voronoi polyhedra of the neighboring TeCl_6_ octahedra share no common faces (Figure 9f). This TeCl_6_ octahedra isolation results in a 0D electronic structure, which agrees well with the results of DFT calculations and optical properties measurements. The two intermediate cases, (Et_2_NH_2_)_2_TeCl_6_ and (Et_3_NH)_2_TeCl_6_, offer a convenient way of probing the effect of slight changes in the outer sphere cation on the nonvalent interactions between the TeCl_6_ complexes. Both compounds have a 2D net of Cl···Cl contacts between TeCl_6_ octahedra (Figure 9d,e), which arrange them in a square net motif. In the case of the smaller Et_2_NH_2_^+^ cation, each TeCl_6_ complex forms four Cl···Cl contacts, which are 3.76 Å apart, with the corresponding Voronoi polyhedron solid angle of 3.59%. The total solid angle of the interacting Cl atoms is 14.36%. The addition of one more ethyl group to the organic cation does not alter the packing of structural units, and similar square layers of interacting TeCl_6_ octahedra form in the structure of (Et_3_NH)_2_TeCl_6_. Moreover, the Te···Te distance demonstrates only a marginal increase from 8.52 to 8.58 Å. Despite this seemingly subtle difference, TeCl_6_ undergoes a slight reorientation that results in Cl···Cl distance elongation from 3.76 to 4.19 Å. This more apparent change leads to a double reduction in the solid angles, from 3.59 to 1.79% (or from a total of 14.36 to 7.16%), which is also associated with reducing the band dispersion of the A_1g_ bands from 0.314 to 0.127 eV for A = Et_2_NH_2_^+^ and Et_3_NH^+^, respectively. This example shows that two nominally electronically 2D structures can have a significantly different degree of orbital overlap, which requires quantification for assessing the properties of the compounds.

Notably, the orbital overlap (i.e., electron coupling) is one of the two components necessary for enabling energy transfer between complexes in hybrid halide compounds, which leads to luminescence quenching. The other requirement is the presence of sufficient spectral overlap according to the Dexter equation.^75,76^ Since spectral overlap is roughly similar in the (Et_*n*_NH_4–*n*_)_2_Sn_1–*x*_Te_*x*_Cl_6_ series due to close Stokes shifts, most of the changes in concentration quenching are due to electron coupling between the metal halide complexes. However, it is important to take into account spectral overlap effects when comparing series with sufficiently different Stokes shifts due to its competing role in luminescence quenching. This interdependence can be illustrated by temperature-dependent PL effects in compounds with high electron coupling. For example, Cs_2_TeCl_6_ exhibits thermal quenching at 200–300 K that occurs as spectral overlap increases with temperature due to exciton–phonon coupling.^77^ At low temperatures, however, there is no significant spectral overlap, thus leading to a “turning off” of PL quenching. On the other hand, PL quenching can be suppressed by strongly diluting Te in a Cs_2_HfCl_6_ matrix, which results in an efficient emission in Cs_2_Hf_0.99_Te_0.01_Cl_6_ at room temperature.^68^

## Conclusions

This report illustrates the difference between electronic and structural dimensionalities and their influence on the optical properties of hybrid halide compounds. Using the (Et_*n*_NH_4*–n*_)_2_Sn_1–*x*_Te_*x*_Cl_6_ series as an example, we showed that interactions between the TeCl_6_^2–^ complexes are strongly dependent on the organic cations. The bulkiest cation, Et_4_N^+^, almost completely shields the complexes from each other, resulting in the lack of an orbital overlap between them as shown by nearly flat bands corresponding to TeCl_6_ A_1g_ orbitals. The near absence of exchange interactions between the complexes results in no concentration quenching effects in the (Et_4_N)_2_Sn_1–*x*_Te_*x*_Cl_6_ solid solutions. On the other hand, the small cation EtNH_3_^+^ offers little shielding and promotes strong interactions between closely arranged TeCl_6_ complexes, leading to a rapid PL concentration quenching in the corresponding Sn/Te solid solutions. The intermediate examples, Et_2_NH_2_^+^ and Et_3_NH^+^, show a decreasing degree of orbital overlap as the cation size increases. Interestingly, the highest PL quantum yields of ∼20% were observed for highly Te-diluted compounds in the (Et_3_NH)_2_Sn_1–*x*_Te_*x*_Cl_6_ series. Since this PLQY value is significantly higher than that in the (Et_4_N)_2_Sn_1–*x*_Te_*x*_Cl_6_ solid solutions, ∼ 4%, the orbital overlap may play a positive role in quantum yields, although further analysis is required to establish this trend unambiguously.

We also demonstrated the application of a simple geometric approach for crystal structure analysis, Voronoi tessellation, for semiquantitative characterization of orbital overlap (and, therefore, band dispersions) in structurally 0D hybrid halide compounds. This automated method allows one to visualize interactions among the isolated complexes and determine their dimensionality and strength. As an example, we demonstrated the correlation between the Cl atoms’ Voronoi polyhedra parameters and optical properties. This method is universal and can be readily extended to other hybrid structures for quick characterization of energy transfer between structurally isolated metal halide complexes. Since many properties of hybrid solid-state materials depend on the degree of the orbital overlap between the isolated complexes, this method offers a fast and simple insight into the properties of the previously synthesized or new materials.

## Experimental Methods

### Synthesis of (Et_*n*_NH_4*–n*_)_2_TeCl _6_(*n* = 1–3) and (Et_3_NH)_2_Te_2_Cl_10_ Single Crystals for X-ray Diffraction (XRD)

For the synthesis of all compounds, TeO_2_ (99.995%) was completely dissolved in excess of concentrated HCl (≥36.5 wt %) on stirring and moderate heating on a hot plate. After dissolution, Et_*n*_NH_3*–n*_ (99%) was slowly added to the solution. In all cases, a stoichiometric ratio TeO_2_:Et_*n*_NH_3*–n*_ of 1:2 was used. Yellow crystals of (EtNH_3_)_2_TeCl_6_ and (Et_3_NH)_2_TeCl_6_ formed upon slow evaporation of solutions at room temperature within several days, while (Et_2_NH_2_)_2_TeCl_6_ crystals formed almost immediately. Two polymorphic modifications of the (Et_3_NH)_2_TeCl_6_ phase, monoclinic and orthorhombic, were found to crystallize simultaneously. Variations in starting molar ratios yielded identical products when ethylamine and diethylamine were used. However, using a 1:1 TeO_2_:Et_3_NH ratio yields a different composition, (Et_3_NH)_2_Te_2_Cl_10_ (Table S28).

### Synthesis of (Et_*n*_NH_4*–n*_)_2_Sn_1–*x*_Te_*x*_Cl_6_ Solid Solutions for Optical Characterization

Four series of (Et_*n*_NH_4*–n*_)_2_Sn_1–*x*_Te_*x*_Cl_6_ (*n* = 1–4) solid solutions with isomorphous substitutions of Sn^4+^ for Te^4+^ were prepared to study the effect of the substitution on the photoluminescent properties. To ensure the starting reagents' complete dissolution (especially sparsely soluble at room temperature SnO_2_) and homogenization, we employed a hydrothermal reaction: (1 – *x*)SnO_2_ + *x*TeO_2_ + 2(Et)_3_NH_3*–n*_/Et_4_NCl → (Et_*n*_NH_4*–n*_)_2_Sn_1–*x*_Te_*x*_Cl_6_ at 190 °C for 3 h in excess of concentrated HCl (Table S28). The reactions yielded crystalline target phases. For Et_3_NH^+^, the hydrothermal reactions yielded transparent solutions, and the target products crystallized after partial solution evaporation. Since the solid-state synthesis route provides more control over the composition, we synthesized several (Et_2_NH_2_)_2_Sn_1–*x*_Te_*x*_Cl_6_ phases via solid-state reactions for comparison with the hydrothermal products. Three (Et_2_NH_2_)_2_Te_1–*x*_Sn_*x*_Cl_6_ phases with *x* = 0.5, 0.1, and 0.05 were prepared by mixing and grounding of (Et_2_NH_2_)_2_SnCl_6_ and (Et_2_NH_2_)TeCl_6_ powders in the corresponding molar ratios. Mixed powders were pressed into pellets using steel dies and annealed for 12 h at 120 °C in a sealed silica tube. After slow cooling (10°/h) to room temperature, pellets were reground and annealed for another 12 h.

### Single-Crystal XRD

Single-crystal X-ray diffraction experiments (Mo Kα radiation) were performed on a Bruker D8 QUEST diffractometer equipped with a PHOTON 100 CMOS area detector at room temperature. Data integration was performed via SAINT-Plus software;^78^ absorption correction was done with the SADABS program.^79^ Structures of new tellurium hybrid chlorides were solved by the intrinsic phasing method (SHELXT^80^ and Olex2^81^) and refined by the full-matrix least-squares method against *F*^2^ in an anisotropic approximation (SHELXL^82^). Hydrogen atoms were placed in geometrically calculated positions if no disorder was presented. Tables S1 and S2 contain crystallographic data on the new hybrid tellurium chlorides with no matches on CCDC.^83^ Note that the structure of (Et_4_N)_2_TeCl_6_ phase was reported earlier.^53^

### Powder XRD

Powder X-ray diffraction patterns for (Et_*n*_NH_4*–n*_)_2_Sn_1–*x*_Te_*x*_Cl_6_ (*n* = 1–4, *x* = 0–1) polycrystalline samples were collected on a Bruker D2 PHASER diffractometer featuring a LYNXEYE XE-T energy-discriminating detector (Cu Kα radiation) over a 5–65° 2θ range.

### Photoluminescence Measurements

Photoluminescence excitation and emission spectra, time-resolved photoluminescence data, and photoluminescence quantum yields were collected at room temperature on an Edinburgh Instruments FS5 spectrofluorometer featuring a 150 W CW ozone-free xenon arc lamp and EPL-375 ps pulsed diode laser excitation sources.

### UV–Vis and Raman Spectroscopy

Diffuse reflectance spectra were collected at room temperature using a Shimadzu UV-2450 (Kyoto, Japan) spectrometer in a wavelength range of 200–800 nm. BaSO_4_ was employed as a nonabsorbing reflectance reference for calibration. Experimental reflection data were transformed into absorption spectra based on the Kubelka–Munk theory, and optical band gaps were extracted. The surface-enhanced Raman spectra were collected using a Renishaw inVia confocal Raman microscope system with a 785 nm laser and with a 9.5 mW laser power (Figure S6).

### Thermogravimetric (TGA) and Differential Thermal Analysis (DTA)

Thermogravimetric analysis (TGA) and differential scanning calorimetry (DSC) were performed on powder samples of pristine (Et_2_NH_2_)_2_TeCl_6_ and (Et_2_NH_2_)SnCl_6_ by using an SDT Q600 thermogravimetric analyzer. Samples were heated from room temperature to 600 °C at a rate of 10 °C/min under a nitrogen flow (100 mL/min) (Figure S6).

### Energy-Dispersive Spectroscopy (EDS)

The elemental analysis of the (Et_*n*_NH_4*–n*_)_2_Sn_1–*x*_Te_*x*_Cl_6_ (*n* = 3 and 4) phases was performed on a Thermo Fisher Scientific Teneo field-emission scanning electron microscope at the Georgia Electron Microscopy facility.

### DFT Calculations

First-principles calculations were performed using density functional theory (DFT) with the Vienna ab initio simulation package (VASP) planewave code,^84,85^ generalized gradient approximation of Perdew, Burke, and Ernzerhof (PBE),^86^ and projector-augmented wave (PAW) method.^87,88^ The initial unit cells were converted to a primitive cell using VESTA software before geometry optimization.^89^ The ground-state geometries at 0 K were optimized by relaxing the cell volume, atomic positions, and cell symmetry until the maximum force on each atom is less than 0.01 eV/Å. Nonspin-polarized calculations were performed, with a 520 eV cutoff energy for the plane wave basis set and 10^–5^ eV energy convergence criteria. The *k*-paths for band structure calculations were generated using the VASPKIT package.^90^ Band structure visualization was performed using the Sumo package.^91^

### Geometric Analysis of Crystal Structures Using Voronoi Tessellation

Crystal structure analysis was performed using the TOPOS 4.0 and ToposPro software packages.^92,93^ The method of intersecting spheres was employed for coordination number determination using the AutoCN program.^74,92−93^ Dirichlet and ADS programs were employed for Voronoi polyhedra construction and topological analysis, respectively.

## Supporting Information Available

Selected crystallographic data and XRD experiment parameters, atomic displacement parameters, bond lengths, bond angles, characteristics of N–H···Cl hydrogen bonds, EDS results and SEM images, average PL lifetimes, PLQYs, parameters of TeCl_6_^2–^ octahedra distortion, synthesis details, Tauc plots, TGA/DTA, and Raman spectra (Tables S1–S28 and Figures S1–S6) (PDF)

## References

[ref1] Snaith H. J. Perovskites: The Emergence of a New Era for Low-Cost, High-Efficiency Solar Cells. J. Phys. Chem. Lett. 2013, 4 (21), 3623–3630. 10.1021/jz4020162.

[ref2] Frost J. M.; Butler K. T.; Brivio F.; Hendon C. H.; van Schilfgaarde M.; Walsh A. Atomistic Origins of High-Performance in Hybrid Halide Perovskite Solar Cells. Nano Lett. 2014, 14 (5), 2584–2590. 10.1021/nl500390f. 24684284 PMC4022647

[ref3] Eames C.; Frost J. M.; Barnes P. R. F.; O’Regan B. C.; Walsh A.; Islam M. S. Ionic Transport in Hybrid Lead Iodide Perovskite Solar Cells. Nat. Commun. 2015, 6 (1), 7497 10.1038/ncomms8497. 26105623 PMC4491179

[ref4] Manser J. S.; Christians J. A.; Kamat P. V. Intriguing Optoelectronic Properties of Metal Halide Perovskites. Chem. Rev. 2016, 116 (21), 12956–13008. 10.1021/acs.chemrev.6b00136. 27327168

[ref5] Sutherland B. R.; Sargent E. H. Perovskite Photonic Sources. Nature Photon 2016, 10 (5), 295–302. 10.1038/nphoton.2016.62.

[ref6] Mao L.; Stoumpos C. C.; Kanatzidis M. G. Two-Dimensional Hybrid Halide Perovskites: Principles and Promises. J. Am. Chem. Soc. 2019, 141 (3), 1171–1190. 10.1021/jacs.8b10851. 30399319

[ref7] Jena A. K.; Kulkarni A.; Miyasaka T. Halide Perovskite Photovoltaics: Background, Status, and Future Prospects. Chem. Rev. 2019, 119 (5), 3036–3103. 10.1021/acs.chemrev.8b00539. 30821144

[ref8] Bibi A.; Lee I.; Nah Y.; Allam O.; Kim H.; Quan L. N.; Tang J.; Walsh A.; Jang S. S.; Sargent E. H.; Kim D. H. Lead-Free Halide Double Perovskites: Toward Stable and Sustainable Optoelectronic Devices. Mater. Today 2021, 49, 123–144. 10.1016/j.mattod.2020.11.026.

[ref9] Song Z.; Zhao J.; Liu Q. Luminescent Perovskites: Recent Advances in Theory and Experiments. Inorg. Chem. Front. 2019, 6 (11), 2969–3011. 10.1039/C9QI00777F.

[ref10] Cortecchia D.; Yin J.; Petrozza A.; Soci C. White Light Emission in Low-Dimensional Perovskites. J. Mater. Chem. C 2019, 7 (17), 4956–4969. 10.1039/C9TC01036J.

[ref11] Zhou G.; Su B.; Huang J.; Zhang Q.; Xia Z. Broad-Band Emission in Metal Halide Perovskites: Mechanism, Materials, and Applications. Materials Science and Engineering: R: Reports 2020, 141, 100548 10.1016/j.mser.2020.100548.

[ref12] Spanopoulos I.; Hadar I.; Ke W.; Guo P.; Mozur E. M.; Morgan E.; Wang S.; Zheng D.; Padgaonkar S.; Manjunatha Reddy G. N.; Weiss E. A.; Hersam M. C.; Seshadri R.; Schaller R. D.; Kanatzidis M. G. Tunable Broad Light Emission from 3D “Hollow” Bromide Perovskites through Defect Engineering. J. Am. Chem. Soc. 2021, 143 (18), 7069–7080. 10.1021/jacs.1c01727. 33905231

[ref13] Dou L.; Yang Y.; You J.; Hong Z.; Chang W. H.; Li G.; Yang Y. Solution-Processed Hybrid Perovskite Photodetectors with High Detectivity. Nat. Commun. 2014, 5 (1), 5404 10.1038/ncomms6404. 25410021

[ref14] Birowosuto M. D.; Cortecchia D.; Drozdowski W.; Brylew K.; Lachmanski W.; Bruno A.; Soci C. X-Ray Scintillation in Lead Halide Perovskite Crystals. Sci. Rep 2016, 6 (1), 37254 10.1038/srep37254. 27849019 PMC5111063

[ref15] Zhao Y.; Zhu K. Organic–Inorganic Hybrid Lead Halide Perovskites for Optoelectronic and Electronic Applications. Chem. Soc. Rev. 2016, 45 (3), 655–689. 10.1039/C4CS00458B. 26645733

[ref16] García de Arquer F. P.; Armin A.; Meredith P.; Sargent E. H. Solution-Processed Semiconductors for next-Generation Photodetectors. Nat. Rev. Mater. 2017, 2 (3), 1–17. 10.1038/natrevmats.2016.100.

[ref17] He Y.; Ke W.; Alexander G. C. B.; McCall K. M.; Chica D. G.; Liu Z.; Hadar I.; Stoumpos C. C.; Wessels B. W.; Kanatzidis M. G. Resolving the Energy of γ-Ray Photons with MAPbI_3_ Single Crystals. ACS Photonics 2018, 5 (10), 4132–4138. 10.1021/acsphotonics.8b00873.

[ref18] Mozur E. M.; Trowbridge J. C.; Maughan A. E.; Gorman M. J.; Brown C. M.; Prisk T. R.; Neilson J. R. Dynamical Phase Transitions and Cation Orientation-Dependent Photoconductivity in CH(NH_2_)_2_PbBr_3_. ACS Materials Lett. 2019, 1 (2), 260–264. 10.1021/acsmaterialslett.9b00209.

[ref19] Kakavelakis G.; Gedda M.; Panagiotopoulos A.; Kymakis E.; Anthopoulos T. D.; Petridis K. Metal Halide Perovskites for High-Energy Radiation Detection. Advanced Science 2020, 7 (22), 2002098 10.1002/advs.202002098. 33240765 PMC7675054

[ref20] Xu L.-J.; Lin X.; He Q.; Worku M.; Ma B. Highly Efficient Eco-Friendly X-Ray Scintillators Based on an Organic Manganese Halide. Nat. Commun. 2020, 11 (1), 4329 10.1038/s41467-020-18119-y. 32859920 PMC7455565

[ref21] Liu R.; Li F.; Zeng F.; Zhao R.; Zheng R. Halide Perovskite X-Ray Detectors: Fundamentals, Progress, and Outlook. Applied Physics Reviews 2024, 11 (2), 021327 10.1063/5.0198695.

[ref22] Gao P.; Grätzel M.; Nazeeruddin M. K. Organohalide Lead Perovskites for Photovoltaic Applications. Energy Environ. Sci. 2014, 7 (8), 2448–2463. 10.1039/C4EE00942H.

[ref23] Brenner T. M.; Egger D. A.; Kronik L.; Hodes G.; Cahen D. Hybrid Organic—Inorganic Perovskites: Low-Cost Semiconductors with Intriguing Charge-Transport Properties. Nat. Rev. Mater. 2016, 1 (1), 1–16. 10.1038/natrevmats.2015.7.

[ref24] Saparov B.; Mitzi D. B. Organic–Inorganic Perovskites: Structural Versatility for Functional Materials Design. Chem. Rev. 2016, 116 (7), 4558–4596. 10.1021/acs.chemrev.5b00715. 27040120

[ref25] Spanopoulos I.; Hadar I.; Ke W.; Tu Q.; Chen M.; Tsai H.; He Y.; Shekhawat G.; Dravid V. P.; Wasielewski M. R.; Mohite A. D.; Stoumpos C. C.; Kanatzidis M. G. Uniaxial Expansion of the 2D Ruddlesden–Popper Perovskite Family for Improved Environmental Stability. J. Am. Chem. Soc. 2019, 141 (13), 5518–5534. 10.1021/jacs.9b01327. 30827098

[ref26] Ke W.; Mao L.; Stoumpos C. C.; Hoffman J.; Spanopoulos I.; Mohite A. D.; Kanatzidis M. G. Compositional and Solvent Engineering in Dion–Jacobson 2D Perovskites Boosts Solar Cell Efficiency and Stability. Adv. Energy Mater. 2019, 9 (10), 1803384 10.1002/aenm.201803384.

[ref27] Spanopoulos I.; Ke W.; Stoumpos C. C.; Schueller E. C.; Kontsevoi O. Y.; Seshadri R.; Kanatzidis M. G. Unraveling the Chemical Nature of the 3D “Hollow” Hybrid Halide Perovskites. J. Am. Chem. Soc. 2018, 140 (17), 5728–5742. 10.1021/jacs.8b01034. 29617127

[ref28] Mozur E. M.; Hope M. A.; Trowbridge J. C.; Halat D. M.; Daemen L. L.; Maughan A. E.; Prisk T. R.; Grey C. P.; Neilson J. R. Cesium Substitution Disrupts Concerted Cation Dynamics in Formamidinium Hybrid Perovskites. Chem. Mater. 2020, 32 (14), 6266–6277. 10.1021/acs.chemmater.0c01862. PMC13235305 42254452

[ref29] Kontos A. G.; Kaltzoglou A.; Arfanis M. K.; McCall K. M.; Stoumpos C. C.; Wessels B. W.; Falaras P.; Kanatzidis M. G. Dynamic Disorder, Band Gap Widening, and Persistent Near-IR Photoluminescence up to At Least 523 K in ASnI_3_ Perovskites (A = Cs^+^, CH_3_NH_3_^+^ and NH_2_–CH=NH_2_^+^). J. Phys. Chem. C 2018, 122 (46), 26353–26361. 10.1021/acs.jpcc.8b10218.

[ref30] Jana A.; Zhumagali S.; Ba Q.; Nissimagoudar A. S.; Kim K. S. Direct Emission from Quartet Excited States Triggered by Upconversion Phenomena in Solid-Phase Synthesized Fluorescent Lead-Free Organic–Inorganic Hybrid Compounds. J. Mater. Chem. A 2019, 7 (46), 26504–26512. 10.1039/C9TA08268A.

[ref31] Fattal H.; Creason T. D.; Delzer C. J.; Yangui A.; Hayward J. P.; Ross B. J.; Du M.-H.; Glatzhofer D. T.; Saparov B. Zero-Dimensional Hybrid Organic–Inorganic Indium Bromide with Blue Emission. Inorg. Chem. 2021, 60 (2), 1045–1054. 10.1021/acs.inorgchem.0c03164. 33397099

[ref32] Morad V.; Yakunin S.; Benin B. M.; Shynkarenko Y.; Grotevent M. J.; Shorubalko I.; Boehme S. C.; Kovalenko M. V. Hybrid 0D Antimony Halides as Air-Stable Luminophores for High-Spatial-Resolution Remote Thermography. Adv. Mater. 2021, 33 (9), 2007355 10.1002/adma.202007355. 33480450 PMC11481058

[ref33] Creason T. D.; Fattal H.; Gilley I. W.; Evans B. N.; Jiang J.; Pachter R.; Glatzhofer D. T.; Saparov B. Stabilized Photoemission from Organic Molecules in Zero-Dimensional Hybrid Zn and Cd Halides. Inorg. Chem. Front. 2022, 9 (23), 6202–6210. 10.1039/D2QI01293F.

[ref34] McWhorter T. M.; Zhang Z.; Creason T. D.; Thomas L.; Du M.-H.; Saparov B. (C_7_H_11_N_2_)_2_MBr_4_ (M = Cu, Zn): X-Ray Sensitive 0D Hybrid Metal Halides with Tunable Broadband Emission. Eur. J. Inorg. Chem. 2022, 2022 (10), e202100954 10.1002/ejic.202100954.

[ref35] Vishnoi P.; Zuo J. L.; Li X.; Binwal D. C.; Wyckoff K. E.; Mao L.; Kautzsch L.; Wu G.; Wilson S. D.; Kanatzidis M. G.; Seshadri R.; Cheetham A. K. Hybrid Layered Double Perovskite Halides of Transition Metals. J. Am. Chem. Soc. 2022, 144 (15), 6661–6666. 10.1021/jacs.1c12760. 35377623

[ref36] Wang S.; Morgan E. E.; Panuganti S.; Mao L.; Vishnoi P.; Wu G.; Liu Q.; Kanatzidis M. G.; Schaller R. D.; Seshadri R. Ligand Control of Structural Diversity in Luminescent Hybrid Copper(I) Iodides. Chem. Mater. 2022, 34 (7), 3206–3216. 10.1021/acs.chemmater.1c04408.

[ref37] Morgan E. E.; Kent G. T.; Zohar A.; O’Dea A.; Wu G.; Cheetham A. K.; Seshadri R. Hybrid and Inorganic Vacancy-Ordered Double Perovskites A_2_WCl_6_. Chem. Mater. 2023, 35 (17), 7032–7038. 10.1021/acs.chemmater.3c01300.

[ref38] McCall K. M.; Morad V.; Benin B. M.; Kovalenko M. V. Efficient Lone-Pair-Driven Luminescence: Structure–Property Relationships in Emissive 5s^2^ Metal Halides. ACS Materials Lett. 2020, 2 (9), 1218–1232. 10.1021/acsmaterialslett.0c00211. PMC7491574 32954359

[ref39] Lyu R.; Moore C. E.; Liu T.; Yu Y.; Wu Y. Predictive Design Model for Low-Dimensional Organic–Inorganic Halide Perovskites Assisted by Machine Learning. J. Am. Chem. Soc. 2021, 143 (32), 12766–12776. 10.1021/jacs.1c05441. 34357756

[ref40] Feng W.; Tan Y.; Yang M.; Jiang Y.; Lei B.-X.; Wang L.; Wu W.-Q. Small Amines Bring Big Benefits to Perovskite-Based Solar Cells and Light-Emitting Diodes. Chem. 2022, 8 (2), 351–383. 10.1016/j.chempr.2021.11.010.

[ref41] Lee J.-W.; Kim D.-H.; Kim H.-S.; Seo S.-W.; Cho S. M.; Park N.-G. Formamidinium and Cesium Hybridization for Photo- and Moisture-Stable Perovskite Solar Cell. Adv. Energy Mater. 2015, 5 (20), 1501310 10.1002/aenm.201501310.

[ref42] Stoumpos C. C.; Cao D. H.; Clark D. J.; Young J.; Rondinelli J. M.; Jang J. I.; Hupp J. T.; Kanatzidis M. G. Ruddlesden–Popper Hybrid Lead Iodide Perovskite 2D Homologous Semiconductors. Chem. Mater. 2016, 28 (8), 2852–2867. 10.1021/acs.chemmater.6b00847.

[ref43] Thouin F.; Valverde-Chávez D. A.; Quarti C.; Cortecchia D.; Bargigia I.; Beljonne D.; Petrozza A.; Silva C.; Srimath Kandada A. R. Phonon Coherences Reveal the Polaronic Character of Excitons in Two-Dimensional Lead Halide Perovskites. Nat. Mater. 2019, 18 (4), 349–356. 10.1038/s41563-018-0262-7. 30643234

[ref44] Mao L.; Chen J.; Vishnoi P.; Cheetham A. K. The Renaissance of Functional Hybrid Transition-Metal Halides. Acc. Mater. Res. 2022, 3 (4), 439–448. 10.1021/accountsmr.1c00270.

[ref45] Han D.; Shi H.; Ming W.; Zhou C.; Ma B.; Saparov B.; Ma Y.-Z.; Chen S.; Du M.-H. Unraveling Luminescence Mechanisms in Zero-Dimensional Halide Perovskites. J. Mater. Chem. C 2018, 6 (24), 6398–6405. 10.1039/C8TC01291A.

[ref46] Maughan A. E.; Ganose A. M.; Scanlon D. O.; Neilson J. R. Perspectives and Design Principles of Vacancy-Ordered Double Perovskite Halide Semiconductors. Chem. Mater. 2019, 31 (4), 1184–1195. 10.1021/acs.chemmater.8b05036.

[ref47] Maughan A. E.; Ganose A. M.; Bordelon M. M.; Miller E. M.; Scanlon D. O.; Neilson J. R. Defect Tolerance to Intolerance in the Vacancy-Ordered Double Perovskite Semiconductors Cs_2_SnI_6_ and Cs_2_TeI_6_. J. Am. Chem. Soc. 2016, 138 (27), 8453–8464. 10.1021/jacs.6b03207. 27284638

[ref48] Lee J. H.; Lee J.-H.; Kong E.-H.; Jang H. M. The Nature of Hydrogen-Bonding Interaction in the Prototypic Hybrid Halide Perovskite, Tetragonal CH_3_NH_3_PbI_3_. Sci. Rep 2016, 6 (1), 21687 10.1038/srep21687. 26892429 PMC4759593

[ref49] Egger D. A. Intermediate Bands in Zero-Dimensional Antimony Halide Perovskites. J. Phys. Chem. Lett. 2018, 9 (16), 4652–4656. 10.1021/acs.jpclett.8b01730. 30052447

[ref50] Nicholas A. D.; Halli R. N.; Garman L. C.; Cahill C. L. Low-Dimensional Hybrid Indium/Antimony Halide Perovskites: Supramolecular Assembly and Electronic Properties. J. Phys. Chem. C 2020, 124 (47), 25686–25700. 10.1021/acs.jpcc.0c07268.

[ref51] Nicholas A. D.; Walusiak B. W.; Garman L. C.; Huda M. N.; Cahill C. L. Impact of Noncovalent Interactions on Structural and Photophysical Properties of Zero-Dimensional Tellurium(IV) Perovskites. J. Mater. Chem. C 2021, 9 (9), 3271–3286. 10.1039/D0TC06000C.

[ref52] Nicholas A. D.; Garman L. C.; Albano N.; Cahill C. L. Insight on Noncovalent Interactions and Orbital Constructs in Low-Dimensional Antimony Halide Perovskites. Phys. Chem. Chem. Phys. 2022, 24 (25), 15305–15320. 10.1039/D2CP01996E. 35703012

[ref53] Bukvetskii B. V.; Sedakova T. V.; Mirochnik A. G. Crystal Structure, Luminescent and Thermochromic Properties of Bis(Tetraethylammonium) Hexachlorotellurate(IV). Russ J. Coord Chem. 2010, 36 (9), 651–656. 10.1134/S1070328410090034.

[ref54] Li Z.; Park J.-S.; Ganose A. M.; Walsh A. From Cubic to Hexagonal: Electronic Trends across Metal Halide Perovskite Polytypes. J. Phys. Chem. C 2023, 127 (26), 12695–12701. 10.1021/acs.jpcc.3c01232.

[ref55] Ghaithan H. M.; Alahmed Z. A.; Qaid S. M. H.; Hezam M.; Aldwayyan A. S. Density Functional Study of Cubic, Tetragonal, and Orthorhombic CsPbBr_3_ Perovskite. ACS Omega 2020, 5 (13), 7468–7480. 10.1021/acsomega.0c00197. 32280890 PMC7144159

[ref56] Hussain M.; Rashid M.; Saeed F.; Bhatti A. S. Spin–Orbit Coupling Effect on Energy Level Splitting and Band Structure Inversion in CsPbBr_3_. J. Mater. Sci. 2021, 56 (1), 528–542. 10.1007/s10853-020-05298-8.

[ref57] Knop O.; Cameron T. S.; James M. A.; Falk M. Bis(Triethylammonium) Hexachlorostannate (IV): Crystal Structure and Hydrogen Bonding. Can. J. Chem. 1981, 59 (16), 2550–2555. 10.1139/v81-367.

[ref58] Knop O.; Cameron T. S.; James M. A.; Falk M. Alkylammonium Hexachlorostannates(IV), (R_n_NH_4–n_)_2_SnCl_6_: Crystal Structure, Infrared Spectrum, and Hydrogen Bonding. Can. J. Chem. 1983, 61 (7), 1620–1646. 10.1139/v83-281.

[ref59] Cameron T. S.; James M. A.; Knop O.; Falk M. Bis(Diethylammonium) Hexachlorostannate(IV), (Et_2_NH_2_)_2_SnCl_6_, and Tris-(Di-n-Propylammonium) Hexachlorostannate(IV) Chloride, (n-Pr_2_NH_2_)_3_(SnCl_6_)Cl: Crystal Structure and Hydrogen Bonding. Can. J. Chem. 1983, 61 (9), 2192–2198. 10.1139/v83-382.

[ref60] Grigoryeva T. F.; Samsonova T. I.; Baidina I. A.; Ivanov E. Yu. Thermal Decomposition of [R_n_NH_4-n_]TeCl_6_ in the Solid State. Izvestiya Sibirskogo otdeleniya Akademii nauk SSSR. Seriya khimicheskikh nauk 1985, 29 (5), 29–34.

[ref61] Sedakova T. V.; Mirochnik A. G. Structure and Luminescent Properties of Complex Compounds of Tellurium(IV) with Ammonium Bases. Opt. Spectrosc. 2015, 119 (1), 54–58. 10.1134/S0030400X15070267.

[ref62] Stufkens D. J. Dynamic Jahn-Teller Effect in the Excited States of SeCl_6_^2–^, SeBr_6_^2–^, TeCl_6_^2–^ and TeBr_6_^2–^: Interpretation of Electronic Absorption and Raman Spectra. Recueil des Travaux Chimiques des Pays-Bas 1970, 89 (11), 1185–1201. 10.1002/recl.19700891109.

[ref63] Ozin G. A.; Voet A. V. The Gas Phase Raman Spectrum and Molecular Structure of Dichlorodibromotellurium(IV) TeCl_2_Br_2_. Novel Penta- and Hexa-Co-Ordinate Mixed Halide Anions of Tellurium(IV). Synthesis and Infrared and Raman Spectra of [Et_4_N]TeCl_2_Br_3_ and [Et_4_N]_2_TeCl_2_Br_4_. Can. J. Chem. 1971, 49 (5), 704–708. 10.1139/v71-118.

[ref64] Clark R. J. H.; Stead M. J. Raman Spectroscopy of the [TeX_6_]^2–^ Ions (X = Cl or Br) at Resonance with Their Lowest ^3^T_1u_ and ^1^T_1u_ States: Evidence for Tetragonal Distortion in These Excited States. Chem. Phys. 1984, 91 (1), 113–118. 10.1016/0301-0104(84)80047-6.

[ref65] Ouasri A.; Elyoubi M. S. D.; Guedira T.; Rhandour A.; Mhiri T.; Daoud A. Synthesis, DTA, IR and Raman Spectra of Penthylenediammonium Hexachlorostannate NH_3_(CH_2_)_5_NH_3_SnCl_6_. Spectrochimica Acta Part A: Molecular and Biomolecular Spectroscopy 2001, 57 (13), 2593–2598. 10.1016/S1386-1425(01)00431-0. 11765786

[ref66] Drummen P. J. H.; Donker H.; Smit W. M. A.; Blasse G. Jahn-Teller Distortion in the Excited State of Tellurium(IV) in Cs_2_MCl_6_ (M = Zr, Sn). Chem. Phys. Lett. 1988, 144 (5), 460–462. 10.1016/0009-2614(88)87296-8.

[ref67] Nikol H.; Becht A.; Vogler A. Photoluminescence of Germanium(II), Tin(II), and Lead(II) Chloride Complexes in Solution. Inorg. Chem. 1992, 31 (15), 3277–3279. 10.1021/ic00041a021.

[ref68] Ackerman J. F. Preparation and Luminescence of Some [K_2_PtCl_6_] Materials. Mater. Res. Bull. 1984, 19 (6), 783–791. 10.1016/0025-5408(84)90036-9.

[ref69] Lufaso M. W.; Woodward P. M. Jahn–Teller Distortions, Cation Ordering and Octahedral Tilting in Perovskites. Acta Cryst. B 2004, 60 (1), 10–20. 10.1107/S0108768103026661. 14734840

[ref70] McCusker J. K.; Rheingold A. L.; Hendrickson D. N. Variable-Temperature Studies of Laser-Initiated ^5^T_2_ → ^1^A_1_ Intersystem Crossing in Spin-Crossover Complexes: Empirical Correlations between Activation Parameters and Ligand Structure in a Series of Polypyridyl Ferrous Complexes. Inorg. Chem. 1996, 35 (7), 2100–2112. 10.1021/ic9507880.

[ref71] Mao L.; Guo P.; Wang S.; Cheetham A. K.; Seshadri R. Design Principles for Enhancing Photoluminescence Quantum Yield in Hybrid Manganese Bromides. J. Am. Chem. Soc. 2020, 142 (31), 13582–13589. 10.1021/jacs.0c06039. 32693585

[ref72] Liao J.-F.; Zhang Z.; Zhou L.; Tang Z.; Xing G. Achieving Near-Unity Red Light Photoluminescence in Antimony Halide Crystals via Polyhedron Regulation. Angew. Chem., Int. Ed. 2024, 63, e202404100 10.1002/anie.202404100. 38616169

[ref73] Serezhkin V. N.; Buslaev Yu. A. Stereochemical Effect of Lone Pair Electrons in Antimony Fluorides. Rus. J. Inorg. Chem. 1997, 42 (7), 1064–1071.

[ref74] Blatov V. A.; Serezhkin V. N. Stereoatomic Model of the Structure of Inorganic and Coordination Compounds. Russ. J. Inorg. Chem. 2000, 45 (Suppl. 2), S105–S222.

[ref75] Dexter D. L.; Schulman J. H. Theory of Concentration Quenching in Inorganic Phosphors. J. Chem. Phys. 1954, 22 (6), 1063–1070. 10.1063/1.1740265.

[ref76] Blasse G.; Grabmaier B. C. Energy Transfer. In Luminescent Materials; Blasse G.; Grabmaier B. C., Eds.; Springer: Berlin, Heidelberg, 1994; pp 91–107. 10.1007/978-3-642-79017-1_5.

[ref77] Blasse G.; Dirksen G. J.; Abriel W. The Influence of Distortion of the Te(IV) Coordination Octahedron on Its Luminescence. Chem. Phys. Lett. 1987, 136 (5), 460–464. 10.1016/0009-2614(87)80287-7.

[ref78] SAINT-Plus (Version 7.68); Bruker AXS Inc.: Madison, Wisconsin, USA. 2007.

[ref79] SADABS Bruker AXS Inc.: Madison, Wisconsin, USA. 2008.

[ref80] Sheldrick G. M. SHELXT – Integrated Space-Group and Crystal-Structure Determination. Acta Cryst. A 2015, 71 (1), 3–8. 10.1107/S2053273314026370. PMC4283466 25537383

[ref81] Dolomanov O. V.; Bourhis L. J.; Gildea R. J.; Howard J. a. K.; Puschmann H. OLEX2: A Complete Structure Solution, Refinement and Analysis Program. J. Appl. Crystallogr. 2009, 42 (2), 339–341. 10.1107/S0021889808042726.

[ref82] Sheldrick G. M. Crystal Structure Refinement with SHELXL. Acta Cryst. C 2015, 71 (1), 3–8. 10.1107/S2053229614024218. PMC4294323 25567568

[ref83] Cambridge Structural Database System; Cambridge Crystallographic Data Centre., 2024.

[ref84] Kresse G.; Furthmüller J. Efficiency of Ab-Initio Total Energy Calculations for Metals and Semiconductors Using a Plane-Wave Basis Set. Comput. Mater. Sci. 1996, 6 (1), 15–50. 10.1016/0927-0256(96)00008-0.

[ref85] Kresse G.; Furthmüller J. Efficient Iterative Schemes for Ab Initio Total-Energy Calculations Using a Plane-Wave Basis Set. Phys. Rev. B 1996, 54 (16), 11169–11186. 10.1103/PhysRevB.54.11169. 9984901

[ref86] Perdew J. P.; Burke K.; Ernzerhof M. Generalized Gradient Approximation Made Simple. Phys. Rev. Lett. 1996, 77 (18), 3865–3868. 10.1103/PhysRevLett.77.3865. 10062328

[ref87] Blöchl P. E. Projector Augmented-Wave Method. Phys. Rev. B 1994, 50 (24), 17953–17979. 10.1103/PhysRevB.50.17953. 9976227

[ref88] Kresse G.; Joubert D. From Ultrasoft Pseudopotentials to the Projector Augmented-Wave Method. Phys. Rev. B 1999, 59 (3), 1758–1775. 10.1103/PhysRevB.59.1758.

[ref89] Momma K.; Izumi F. VESTA 3 for Three-Dimensional Visualization of Crystal, Volumetric and Morphology Data. J. Appl. Crystallogr. 2011, 44 (6), 1272–1276. 10.1107/S0021889811038970.

[ref90] Wang V.; Xu N.; Liu J.-C.; Tang G.; Geng W.-T. VASPKIT: A User-Friendly Interface Facilitating High-Throughput Computing and Analysis Using VASP Code. Comput. Phys. Commun. 2021, 267, 108033 10.1016/j.cpc.2021.108033.

[ref91] M Ganose A.; J Jackson A.; O Scanlon D. Sumo: Command-Line Tools for Plotting and Analysis of Periodic *ab Initio* Calculations. J. Open Source Software 2018, 3 (28), 717 10.21105/joss.00717.

[ref92] Blatov V. A.; Shevchenko A. P.; Serezhkin V. N. TOPOS3.2: A New Version of the Program Package for Multipurpose Crystal-Chemical Analysis. J. Appl. Crystallogr. 2000, 33 (4), 1193–1193. 10.1107/S0021889800007202.

[ref200] Blatov V. A. Nanocluster Analysis of Intermetallic Structures with the Program Package TOPOS. Struct. Chem. 2012, 23, 955–963. 10.1007/s11224-012-0013-3.

[ref93] Blatov V. A.; Shevchenko A. P.; Proserpio D. M. Applied Topological Analysis of Crystal Structures with the Program Package ToposPro. Cryst. Growth Des. 2014, 14 (7), 3576–3586. 10.1021/cg500498k.

