# Supplementary material for: Application of Voronoi Polyhedra for Analysis of Electronic Dimensionality in Emissive Halide Materials
Source: J Am Chem Soc. 2024 Dec 10;146(51):35449–61. doi: 10.1021/jacs.4c14554 (PMC11673566; doi:10.1021/jacs.4c14554)
Supplement: Supplementary file 1 — ja4c14554_si_001.pdf [file ja4c14554_si_001.pdf]

Supporting Information for

Application of Voronoi Polyhedra for Analysis of Electronic Dimensionality in Emissive Halide Materials

Sergei A. Novikov, Hope A. Long, Aleksandra D. Valueva, and Vladislav V. Klepov[1]

Department of Chemistry, University of Georgia, Athens, Georgia, 30602, USA

[1] Corresponding author

email: klepov@uga.edu (V. V. Klepov)

Table of Contents

Table S1-S2. Selected crystallographic data and XRD experiment parameters S3-S4

Table S3-S7. Fractional Atomic Coordinates and Equivalent Isotropic Displacement Parameters S5-S7

Table S8-S12. Anisotropic Displacement Parameters S8-S10

Table S13-S17. Bond Lengths S10-S12

Table S18-S22. Bond Angles S12-S14

Table S23. Characteristics of N-H···Cl hydrogen bonds S15

Figure S1 Different dimensionalities of clusters built on N-H···Cl hydrogen bonds S15

Figure S2. Bands dispersion in $(Et_nNH_{4-n})_2Sn_{1-x}Te_xCl_6$ samples S16

Table S24. EDS results S16

Figure S3 EDS results and SEM images S17

Figure S4. PL peak width (a) and asymmetry (b) in $(Et_nNH_{4-n})_2Sn_{1-x}Te_xCl_6$ samples S18

Figure S5. Maximum PL intensity in $(Et_nNH_{4-n})_2Sn_{1-x}Te_xCl_6$ samples S18

Table S25. Average PL lifetimes S19

Table S26. PLQY S19

Table S27. Parameters of $TeCl_6^{2-}$ octahedra distortion S20

Table S28. Synthesis S20

Figure S6. Tauc plots, TGA/DTA, Raman spectra S21

References S22

Table S1. Selected crystallographic data and XRD experiment parameters for $(Et_nNH_{4-n})_2TeCl_6$ phases (n = 1–3).

Phase	$(EtNH_3)_2TeCl_6$	$(Et_2NH_2)_2TeCl_6$	m-$(Et_3NH)_2TeCl_6$
Formula weight	422.41	488.59	544.69
Temperature, K	298	300.00	298.00
Crystal system	trigonal	monoclinic	monoclinic
Space group	$P\bar{3}m1$	$P2_1/n$	$P2_1/n$
a, Å	7.4216(7)	9.1743(4)	9.9605(9)
b, Å	7.4216(7)	10.7653(5)	10.8251(11)
c, Å	8.3276(12)	9.8404(5)	10.8083(11)
α, °	90	90	90
β, °	90	92.1340(10)	100.434(3)
γ, °	120	90	90
Volume, $Å^3$	397.23(9)	971.20(8)	1146.12(19)
Z	1	2	2
ρcalc, g/cm^3	1.766	1.671	1.578
μ, mm-1	2.849	2.342	1.994
F(000)	198	480.0	544.0
Crystal size, mm^3	0.1 × 0.08 × 0.03	0.15 × 0.1 × 0.02	0.06 × 0.05× 0.01
2Θ range for data collection, °	6.34 - 57.322	5.61 - 61.274	5.12 - 49.938
Radiation	MoKα (λ = 0.71073)	MoKα (λ = 0.71073)	MoKα (λ = 0.71073)
Index ranges	$-10 \leq h \leq 10$, $-9 \leq k \leq 10$, $-11 \leq l \leq 11$	$-13 \leq h \leq 13$, $-15 \leq k \leq 14$, $-14 \leq l \leq 14$	$-11 \leq h \leq 11$, $-12 \leq k \leq 12$, $-12 \leq l \leq 12$
Reflections collected	6290	33187	12693
Independent reflections	419 R_{int} = 0.0300	2979 R_{int} = 0.0284	2011 R_{int} = 0.0953
Data, restraints, parameters	419,0,22	2979,0,82	2011,0,101
Goodness-of-fit on, F^2	1.111	1.237	1.027
Final R indexes, I≥2σ (I)	R_1 = 0.0308, wR_2 = 0.0814	R_1 = 0.0403, wR_2 = 0.1110	R_1 = 0.0344, wR_2 = 0.0467
Final R indexes, all data	R_1 = 0.0367, wR_2 = 0.0844	R_1 = 0.0515, wR_2 = 0.1312	R_1 = 0.0708, wR_2 = 0.0540
Largest diff. peak, hole, e/$Å^{-3}$	0.55/-0.72	1.41/-1.77	0.42/-0.35

Table S2. Selected crystallographic data and XRD experiment parameters for o-$(Et_3NH)_2TeCl_6$ and $(Et_3NH)_2Te_2Cl_{10}$ phases.

**Phase**	**o-$(Et_3NH)_2TeCl_6$**	**$(Et_3NH)_2Te_2Cl_{10}$**
**Formula weight**	544.69	810.06
**Temperature, K**	298	298.00
**Crystal system**	orthorhombic	monoclinic
**Space group**	*Pbca*	$P2_1/n$
**a, Å**	19.355(9)	10.202(5)
**b, Å**	18.716(10)	14.085(7)
**c, Å**	26.594(13)	10.650(6)
**α, °**	90	90
**β, °**	90	92.366(17)
**γ, °**	90	90
**Volume, $Å^3$**	9634(8)	1529.1(13)
**Z**	16	2
**ρcalc, g/cm^3**	1.502	1.759
**μ, mm-1**	1.898	2.785
**F(000)**	4352	776.0
**Crystal size, mm^3**	0.07 × 0.05 × 0.02	0.07 × 0.05× 0.02
**2Θ range for data collection, °**	3.392 - 41.654	4.798 - 75.832
**Radiation**	MoKα (λ = 0.71073)	MoKα (λ = 0.71073)
**Index ranges**	$-19 \leq h \leq 18$, $-18 \leq k \leq 18$, $-24 \leq l \leq 26$	$-14 \leq h \leq 13$, $-23 \leq k \leq 24$, $-15 \leq l \leq 15$
**Reflections collected**	56778	38763
**Independent reflections**	4991 $R_{int} = 0.1489$	5725 $R_{int} = 0.2630$
**Data, restraints, parameters**	4991, 0, 391	5725, 0, 122
**Goodness-of-fit on, F^2**	1.023	1.014
**Final R indexes, I≥2σ (I)**	$R_1 = 0.0434$, $wR_2 = 0.0709$	$R_1 = 0.1030$, $wR_2 = 0.2731$
**Final R indexes, all data**	$R_1 = 0.0803$, $wR_2 = 0.0814$	$R_1 = 0.2793$, $wR_2 = 0.3699$
**Largest diff. peak, hole, e/$Å^{-3}$**	0.31/-0.34	1.65/-2.01

Table S3. Fractional Atomic Coordinates (×10^4) and Equivalent Isotropic Displacement Parameters (Å^2×10^3) for $(EtNH_3)_2TeCl_6$. U_{eq} is defined as 1/3 of the trace of the orthogonalised U_{IJ} tensor.

Atom	*x*	*y*	*z*	U(eq)
Te1	10000	10000	5000	58.2(4)
Cl1	8359.5(15)	6719(3)	3324(3)	91.1(7)
N1	6666.67	3333.33	6600(12)	91(4)
C1	6666.67	3333.33	8270(18)	153(11)
C2	6080(30)	2150(50)	9680(30)	145(14)

Table S4. Fractional Atomic Coordinates (×10^4) and Equivalent Isotropic Displacement Parameters (Å^2×10^3) for $(Et_2NH_2)_2TeCl_6$. U_{eq} is defined as 1/3 of the trace of the orthogonalised U_{IJ} tensor.

Atom	*x*	*y*	*z*	U(eq)
Te1	5000	5000	5000	29.52(9)
Cl1	5633.1(10)	3325.3(9)	6734.7(8)	60.6(2)
Cl2	3377.8(8)	6048.1(7)	6692.1(7)	47.36(17)
Cl3	7143.4(8)	6263.2(9)	5905.9(9)	61.1(2)
N1	5759(3)	7873(2)	8436(2)	43.9(5)
C1	6469(4)	6139(4)	9938(4)	60.9(8)
C2	5478(4)	7229(3)	9739(3)	50.4(7)
C3	4818(4)	8962(3)	8118(4)	56.3(8)
C4	5146(5)	9518(4)	6773(4)	70.6(10)

Table S5. Fractional Atomic Coordinates (×10^4) and Equivalent Isotropic Displacement Parameters (Å^2×10^3) for m-$(Et_3NH)_2TeCl_6$. U_{eq} is defined as 1/3 of the trace of the orthogonalised U_{IJ} tensor.

Atom	*x*	*y*	*z*	U(eq)
Te1	5000	5000	5000	31.07(14)
Cl2	3698.4(11)	6957.8(11)	4308.8(13)	64.3(4)
Cl1	5636.7(13)	5872.5(13)	7186.3(11)	75.0(4)
Cl3	2896.9(10)	4002.5(9)	5609.6(11)	46.0(3)

N1	4048(3)	1500(3)	7267(3)	39.2(9)
C5	2833(4)	894(4)	6478(4)	48.1(11)
C4	5169(5)	-526(4)	8010(5)	66.7(15)
C2	4653(5)	2879(4)	9151(4)	67.9(15)
C3	5318(4)	732(4)	7466(4)	49.9(12)
C6	3082(5)	498(4)	5212(4)	66.1(14)
C1	3661(5)	1988(4)	8453(4)	55.1(12)

Table S6. Fractional Atomic Coordinates (×10^4) and Equivalent Isotropic Displacement Parameters (Å^2×10^3) for o-$(Et_3NH)_2TeCl_6$. U_{eq} is defined as 1/3 of the trace of the orthogonalised U_{IJ} tensor.

**Atom**	***x***	***y***	***z***	**U(eq)**
Te2	5134.6(2)	4958.1(3)	7709.8(2)	62.62(18)
Cl0A	4556.2(12)	6207.4(12)	7827.9(10)	95.8(8)
Cl12	5698.8(12)	5362.1(15)	6805.5(9)	101.3(9)
Cl10	4088.3(11)	4532.8(12)	7266.7(10)	92.3(8)
Cl9	6216.2(12)	5438.3(13)	8160.1(10)	104.4(9)
Cl8	4646.8(13)	4609.0(14)	8506.7(9)	101.2(8)
Cl11	5640.2(12)	3746.2(12)	7645.8(10)	96.4(8)
Te1	7569.5(3)	8081.8(3)	5155.2(2)	60.99(18)
Cl2	6859.1(13)	6936.2(13)	5041.3(10)	106.4(9)
Cl6	8266.6(12)	9210.1(12)	5253.6(9)	88.1(7)
Cl3	8682.1(12)	7372.6(12)	4907.6(10)	97.0(8)
Cl5	6521.4(12)	8743.7(13)	5402.0(9)	94.4(8)
Cl1	7358.8(12)	8385.2(13)	4227.7(8)	92.7(8)
Cl4	7771.4(14)	7759.3(13)	6057.7(8)	98.8(8)
N3	7352(3)	5331(4)	6686(3)	74(2)
C17	7717(5)	5689(5)	7108(3)	97(3)
C13	7571(5)	4555(5)	6630(3)	93(3)
C14	7396(5)	4091(5)	7064(3)	104(3)
C15	7386(5)	5719(5)	6184(3)	91(3)
C16	8096(5)	5781(5)	5972(4)	119(4)
C18	7471(5)	6442(5)	7210(3)	111(3)
N1	5252(5)	7284(5)	8651(3)	108(3)
C4	6054(7)	7492(6)	7965(5)	164(6)
C3	5866(8)	7582(9)	8411(7)	237(10)
C5	5437(9)	6982(9)	9149(7)	204(7)
C2	4773(7)	8452(7)	8918(5)	187(6)
C6	5063(6)	6401(7)	9335(4)	146(5)

C1	4649(7)	7758(8)	8660(5)	158(5)
N2	4928(5)	6738(6)	6103(4)	130(3)
C11	5181(9)	6518(9)	5655(6)	218(9)
C10	5816(6)	7516(6)	6507(5)	165(5)
C7	4308(9)	6666(11)	6272(7)	312(16)
C9	5203(10)	7461(10)	6219(8)	278(12)
C12	5215(7)	5840(7)	5436(5)	160(5)
C8	3883(6)	6559(8)	6597(5)	183(6)
N4	7128(6)	4341(7)	9068(5)	173(5)
C19	6984(9)	3728(8)	8856(5)	203(8)
C22	8329(8)	4532(7)	9147(5)	188(6)
C24	6079(6)	4566(6)	9592(4)	125(4)
C20	7236(6)	3366(6)	8424(4)	150(5)
C21	7819(7)	4760(7)	8828(6)	160(5)
C23	6821(8)	4671(6)	9482(4)	140(5)

Table S7. Fractional Atomic Coordinates (×10^4) and Equivalent Isotropic Displacement Parameters (Å^2×10^3) for $(Et_3NH)_2Te_2Cl_{10}$. U_{eq} is defined as 1/3 of the trace of the orthogonalised U_{IJ} tensor.

**Atom**	***x***	***y***	***z***	**U(eq)**
Te1	6331.5(8)	5441.8(6)	6414.4(8)	49.2(6)
Cl3	4418(5)	5448(3)	7847(5)	79.9(13)
Cl4	8219(4)	5438(4)	4983(5)	79.2(13)
Cl2	5482(4)	3681(3)	5469(4)	67.4(10)
Cl1	7655(6)	4666(4)	7992(5)	100.9(18)
Cl5	6988(4)	6991(3)	7188(4)	80.4(13)
N1	1815(15)	7012(13)	6856(12)	85(4)
C5	2430(20)	7814(17)	7506(17)	96(6)
C6	3470(30)	8370(20)	6740(20)	125(9)
C3	1210(30)	6190(20)	8880(20)	122(9)
C2	1260(20)	7170(16)	5540(17)	92(6)
C4	790(20)	6533(19)	7660(20)	106(7)
C1	190(20)	7886(18)	5470(20)	107(7)

Table S8. Anisotropic Displacement Parameters (Å^2×10^3) for $(EtNH_3)_2TeCl_6$. The anisotropic displacement factor exponent takes the form: $-2\pi^2[h^2a\times{}^2U_{11}+2hka\times b\times U_{12}+\ldots]$.

Atom	U_{11}	U_{22}	U_{33}	U_{23}	U_{13}	U_{12}
Te1	34.6(4)	34.6(4)	105.5(8)	0	0	17.30(18)
Cl1	82.5(10)	52.7(9)	128.2(15)	-33.0(10)	-16.5(5)	26.4(5)
N1	101(6)	101(6)	71(6)	0	0	51(3)
C1	186(17)	186(17)	87(11)	0	0	93(9)

Table S9. Anisotropic Displacement Parameters (Å^2×10^3) for $(Et_2NH_2)_2TeCl_6$. The anisotropic displacement factor exponent takes the form: $-2\pi^2[h^2a\times{}^2U_{11}+2hka\times b\times U_{12}+\ldots]$.

Atom	U_{11}	U_{22}	U_{33}	U_{23}	U_{13}	U_{12}
Te1	27.22(12)	34.74(13)	26.56(12)	-2.04(7)	0.38(7)	1.29(7)
Cl1	63.7(5)	65.8(5)	53.2(4)	24.6(4)	14.8(3)	20.8(4)
Cl2	42.7(3)	52.5(4)	47.7(4)	-11.3(3)	12.7(3)	1.1(3)
Cl3	39.9(3)	86.3(6)	57.6(4)	-29.1(4)	10.6(3)	-22.2(4)
N1	40.3(11)	53.6(14)	37.9(11)	-6.9(10)	5.4(9)	-4.0(10)
C1	57.8(19)	63(2)	62(2)	11.3(16)	7.0(15)	3.3(16)
C2	48.8(15)	65(2)	37.5(13)	-2.0(13)	9.5(11)	2.2(14)
C3	64(2)	50.0(17)	55.4(18)	-0.1(14)	11.2(15)	4.5(15)
C4	76(3)	72(2)	64(2)	17(2)	0.4(19)	-10(2)

Table S10. Anisotropic Displacement Parameters (Å^2×10^3) for m-$(Et_3NH)_2TeCl_6$. The anisotropic displacement factor exponent takes the form: $-2\pi^2[h^2a\times{}^2U_{11}+2hka\times b\times U_{12}+\ldots]$.

Atom	U_{11}	U_{22}	U_{33}	U_{23}	U_{13}	U_{12}
Te1	29.7(2)	28.4(2)	36.4(2)	4.6(2)	9.20(14)	-2.2(2)
Cl2	46.9(7)	48.5(7)	102.5(11)	28.7(7)	26.5(7)	11.8(6)
Cl1	85.2(10)	89.3(10)	49.6(8)	-17.2(7)	9.9(7)	-21.6(8)
Cl3	34.8(6)	42.0(6)	64.3(8)	8.4(6)	17.3(5)	-1.3(5)
N1	43(2)	38(2)	39(2)	8.6(17)	11.6(17)	-1.2(17)
C5	43(3)	45(3)	54(3)	4(2)	2(2)	-3(2)
C4	57(3)	66(3)	78(4)	29(3)	15(3)	14(3)
C2	85(4)	58(3)	58(4)	-7(3)	3(3)	1(3)
C3	40(3)	61(3)	50(3)	11(3)	13(2)	0(2)
C6	74(3)	63(3)	57(4)	-11(3)	1(3)	-3(3)
C1	70(3)	53(3)	45(3)	2(2)	17(3)	4(3)

Table S11. Anisotropic Displacement Parameters (Å^2×10^3) for o-$(Et_3NH)_2TeCl_6$. The anisotropic displacement factor exponent takes the form: $-2\pi^2[h^2a \times {}^2U_{11}+2hka \times b \times U_{12}+\ldots]$.

Atom	U_{11}	U_{22}	U_{33}	U_{23}	U_{13}	U_{12}
Te2	50.8(3)	62.7(4)	74.5(4)	6.5(3)	-5.4(3)	-4.9(3)
Cl0A	81.2(17)	76.2(17)	130(2)	-16.0(15)	-26.5(15)	7.9(13)
Cl12	69.9(16)	143(2)	90.9(18)	33.2(16)	5.3(14)	7.5(15)
Cl10	61.7(14)	88.3(18)	127(2)	-25.5(16)	-12.8(15)	-4.3(12)
Cl9	92.4(18)	100(2)	121(2)	29.4(16)	-40.7(16)	-28.7(15)
Cl8	106(2)	108(2)	89.3(18)	11.0(15)	19.6(15)	-17.2(16)
Cl11	82.6(16)	77.5(17)	129(2)	9.8(16)	0.8(15)	14.0(13)
Te1	67.6(4)	50.6(3)	64.8(3)	-1.3(3)	7.1(3)	-1.5(3)
Cl2	104.7(19)	80.4(17)	134(2)	-17.6(17)	26.7(17)	-33.0(15)
Cl6	97.5(18)	68.8(16)	98.0(18)	-1.7(13)	-2.7(14)	-15.5(13)
Cl3	90.1(18)	81.1(17)	120(2)	-3.5(15)	6.3(16)	20.6(14)
Cl5	88.0(17)	89.7(18)	105.4(19)	-3.0(15)	18.8(15)	17.8(15)
Cl1	95.8(18)	112(2)	70.8(15)	3.6(13)	-3.4(14)	-6.3(15)
Cl4	138(2)	83.6(17)	74.2(16)	13.5(14)	2.6(15)	3.7(16)
N3	60(5)	85(6)	77(5)	4(4)	5(4)	6(4)
C17	98(8)	108(9)	85(7)	-6(6)	-21(6)	-6(7)
C13	99(8)	86(7)	93(7)	12(6)	11(6)	19(6)
C14	119(9)	85(7)	109(8)	18(6)	13(7)	-12(6)
C15	110(9)	80(7)	84(7)	12(6)	8(6)	10(6)
C16	126(10)	108(9)	124(9)	12(7)	47(8)	15(7)
C18	136(9)	82(7)	114(8)	-20(6)	-4(7)	-1(7)
N1	129(8)	118(8)	77(6)	-20(6)	11(6)	-41(7)
C4	175(13)	131(11)	185(15)	-26(10)	81(12)	-58(9)
C3	179(15)	280(20)	250(20)	-132(17)	141(15)	-141(14)
C5	260(20)	184(17)	171(17)	-23(14)	-54(15)	-56(15)
C2	201(15)	132(12)	229(16)	-39(12)	57(12)	9(11)
C6	166(13)	153(13)	120(10)	28(9)	-1(9)	5(10)
C1	114(11)	154(13)	207(16)	-9(12)	6(10)	16(10)
N2	108(8)	149(10)	132(9)	-40(7)	24(7)	-17(7)
C11	290(20)	220(20)	145(15)	5(13)	61(15)	114(18)
C10	140(12)	119(11)	236(17)	22(10)	-16(11)	-26(9)
C7	145(15)	420(30)	370(30)	210(20)	170(19)	72(18)
C9	260(20)	200(20)	380(30)	-37(18)	-140(20)	-61(18)
C12	177(13)	174(14)	128(11)	-40(10)	4(9)	14(11)
C8	88(10)	310(20)	149(12)	46(12)	-11(9)	4(11)
N4	115(9)	191(13)	213(13)	-112(11)	32(9)	-11(8)
C19	330(20)	141(13)	141(13)	-45(11)	64(14)	-85(14)
C22	222(18)	179(15)	164(14)	-32(11)	-36(12)	49(13)

C24	119(10)	117(10)	141(10)	10(8)	11(8)	6(8)
C20	152(11)	144(11)	154(11)	-28(9)	-38(9)	64(9)
C21	140(13)	142(13)	197(16)	-21(11)	-8(12)	22(10)
C23	220(16)	107(10)	93(9)	-8(7)	13(10)	-23(11)

Table S12. Anisotropic Displacement Parameters ($Å^2 \times 10^3$) for $(Et_3NH)_2Te_2Cl_{10}$. The anisotropic displacement factor exponent takes the form: $-2\pi^2[h^2a\times{}^2U_{11}+2hka\times b\times U_{12}+\ldots]$.

**Atom**	**U_{11}**	**U_{22}**	**U_{33}**	**U_{23}**	**U_{13}**	**U_{12}**
Te1	42.8(7)	44.9(7)	60.1(8)	-2.4(4)	4.9(4)	-0.6(4)
Cl3	79(3)	82(3)	82(3)	-5(2)	33(2)	-4(2)
Cl4	54(2)	85(3)	100(3)	-11(2)	26(2)	-4.2(19)
Cl2	70(2)	46(2)	87(2)	0.4(18)	15.2(17)	5.8(17)
Cl1	105(4)	92(4)	103(3)	25(3)	-30(3)	6(3)
Cl5	77(3)	57(2)	108(3)	-23(2)	12(2)	-17(2)
N1	85(10)	95(12)	77(8)	-6(9)	3(7)	18(9)
C5	97(15)	100(18)	90(13)	-11(12)	-6(11)	6(13)
C6	125(19)	120(20)	134(19)	-29(16)	58(16)	-13(16)
C3	129(19)	130(20)	106(15)	11(16)	-2(14)	32(17)
C2	109(15)	84(15)	84(11)	5(10)	5(10)	-1(12)
C4	92(14)	107(19)	119(16)	30(13)	26(12)	-7(12)
C1	98(15)	106(18)	116(15)	38(14)	3(12)	20(14)

Table S13. Bond Lengths for $(EtNH_3)_2TeCl_6$ phase.

**Atom**	**Atom**	**Length, Å**	**Atom**	**Atom**	**Length, Å**
Te1	Cl1[1]	2.5290(17)	N1	C1	1.391(15)
Te1	Cl1	2.5290(17)	C1	C2	1.402(18)
Te1	Cl1[2]	2.5290(17)	C1	C2[6]	1.402(18)
Te1	Cl1[3]	2.5290(17)	C1	C2[7]	1.402(18)
Te1	Cl1[4]	2.5290(17)	C2	C2[7]	1.32(6)
Te1	Cl1[5]	2.5290(17)	C2	C2[6]	1.32(6)

[1]1-Y+X,+X,1-Z; [2]+Y,1-X+Y,1-Z; [3]2-Y,1+X-Y,+Z; [4]2-X,2-Y,1-Z; [5]1+Y-X,2-X,+Z; [6]1-Y,+X-Y,+Z; [7]1+Y-X,1-X,+Z

Table S14. Bond Lengths for $(Et_2NH_2)_2TeCl_6$ phase.

Atom	Atom	Length, Å	Atom	Atom	Length, Å
Te1	Cl1[1]	2.5353(7)	Te1	Cl3[1]	2.5259(7)
Te1	Cl1	2.5353(7)	N1	C2	1.489(4)
Te1	Cl2	2.5387(6)	N1	C3	1.483(4)
Te1	Cl2[1]	2.5388(6)	C1	C2	1.493(5)
Te1	Cl3	2.5259(7)	C3	C4	1.494(5)

[1]1-X,1-Y,1-Z

Table S15. Bond Lengths for m-$(Et_3NH)_2TeCl_6$ phase.

Atom	Atom	Length, Å	Atom	Atom	Length, Å
Te1	Cl21	2.5258(11)	N1	C5	1.499(5)
Te1	Cl2	2.5258(11)	N1	C3	1.496(5)
Te1	Cl1	2.5170(12)	N1	C1	1.500(5)
Te1	Cl11	2.5170(12)	C5	C6	1.496(5)
Te1	Cl3	2.5472(9)	C4	C3	1.502(5)
Te1	Cl31	2.5472(9)	C2	C1	1.485(6)

[1]1-X,1-Y,1-Z

Table S16. Bond Lengths for o-$(Et_3NH)_2TeCl_6$ phase.

Atom	Atom	Length, Å	Atom	Atom	Length, Å
Te2	Cl0A	2.611(3)	N1	C3	1.459(13)
Te2	Cl12	2.748(3)	N1	C5	1.485(16)
Te2	Cl10	2.475(2)	N1	C1	1.468(13)
Te2	Cl9	2.574(2)	C4	C3	1.254(14)
Te2	Cl8	2.410(3)	C5	C6	1.397(15)
Te2	Cl11	2.476(3)	C2	C1	1.489(14)
Te1	Cl2	2.565(3)	N2	C11	1.354(14)
Te1	Cl6	2.520(2)	N2	C7	1.287(15)
Te1	Cl3	2.614(2)	N2	C9	1.486(16)
Te1	Cl5	2.466(2)	C11	C12	1.398(16)
Te1	Cl1	2.564(3)	C10	C9	1.417(16)
Te1	Cl4	2.506(3)	C7	C8	1.211(15)
N3	C17	1.485(9)	N4	C19	1.308(13)
N3	C13	1.521(9)	N4	C21	1.676(15)
N3	C15	1.521(9)	N4	C23	1.396(12)

C17	C18	1.512(11)	C19	C20	1.420(14)
C13	C14	1.484(10)	C22	C21	1.370(14)
C15	C16	1.490(11)	C24	C23	1.479(14)
Te2	Cl0A	2.611(3)	N1	C3	1.459(13)

Table S17. Bond Lengths for $(Et_3NH)_2Te_2Cl_{10}$ phase.

**Atom**	**Atom**	**Length, Å**	**Atom**	**Atom**	**Length, Å**
Te1	Cl3	2.527(4)	N1	C5	1.45(3)
Te1	Cl4	2.505(4)	N1	C2	1.51(2)
Te1	Cl2	2.801(4)	N1	C4	1.54(2)
Te1	Cl2^1	2.944(4)	C5	C6	1.57(3)
Te1	Cl1	2.378(5)	C3	C4	1.43(3)
Te1	Cl5	2.417(4)	C2	C1	1.49(3)

^{1}1-X,1-Y,1-Z

Table S18. Bond Angles for $(EtNH_3)_2TeCl_6$ phase.

**Atom**	**Atom**	**Atom**	**Angle/°**	**Atom**	**Atom**	**Angle/°**
Cl1^1	Te1	Cl1^2	180	Cl1^1	Te1	Cl1^4
Cl1^3	Te1	Cl1^4	87.54(8)	Cl1^2	Te1	Cl1^4
Cl1^3	Te1	Cl1^5	92.46(8)	Cl1^4	Te1	Cl1^5
Cl1	Te1	Cl1^4	92.46(8)	N1	C1	C2^6
Cl1^2	Te1	Cl1^5	87.54(8)	N1	C1	C2
Cl1	Te1	Cl1^5	87.54(8)	N1	C1	C2^7
Cl1^1	Te1	Cl1^3	92.46(8)	C2^6	C1	C2^7
Cl1^1	Te1	Cl1	87.54(8)	C2^6	C1	C2
Cl1^2	Te1	Cl1^3	87.54(8)	C2	C1	C2^7
Cl1^1	Te1	Cl1^5	92.46(8)	C2^6	C2	C1
Cl1	Te1	Cl1^3	180	C2^7	C2	C1
Cl1^2	Te1	Cl1	92.46(8)	C2^7	C2	C2^6

^{1}1-Y+X,+X,1-Z; ^{2}1+Y-X,2-X,+Z; ^{3}2-X,2-Y,1-Z; ^{4}2-Y,1+X-Y,+Z; ^{5}+Y,1-X+Y,1-Z; ^{6}1-Y,+X-Y,+Z; ^{7}1+Y-X,1-X,+Z

Table S19. Bond Angles for $(Et_2NH_2)_2TeCl_6$ phase.

Atom	Atom	Atom	Angle/°	Atom	Atom	Angle/°
Cl1[1]	Te1	Cl1	180	Cl3	Te1	Cl1
Cl1	Te1	Cl2	89.98(3)	Cl3	Te1	Cl2[1]
Cl1	Te1	Cl2[1]	90.02(3)	Cl3[1]	Te1	Cl2
Cl1[1]	Te1	Cl2	90.02(3)	Cl3	Te1	Cl2
Cl1[1]	Te1	Cl2[1]	89.98(3)	Cl3[1]	Te1	Cl2[1]
Cl2	Te1	Cl2[1]	180	Cl3	Te1	Cl3[1]
Cl3[1]	Te1	Cl1[1]	89.39(3)	C3	N1	C2
Cl3[1]	Te1	Cl1	90.61(3)	N1	C2	C1
Cl3	Te1	Cl1[1]	90.61(3)	N1	C3	C4

[1]1-X,1-Y,1-Z

Table S20. Bond Angles for m-$(Et_3NH)_2TeCl_6$ phase.

Atom	Atom	Atom	Angle/°	Atom	Atom	Angle/°
Cl2[1]	Te1	Cl2	180.00(4)	Cl1[1]	Te1	Cl3[1]
Cl2	Te1	Cl3	91.72(3)	Cl1	Te1	Cl3[1]
Cl2[1]	Te1	Cl3[1]	91.72(3)	Cl1	Te1	Cl3
Cl2[1]	Te1	Cl3	88.28(3)	Cl3[1]	Te1	Cl3
Cl2	Te1	Cl3[1]	88.28(3)	C5	N1	C1
Cl1[1]	Te1	Cl2	90.49(5)	C3	N1	C5
Cl1[1]	Te1	Cl2[1]	89.51(5)	C3	N1	C1
Cl1	Te1	Cl2	89.51(5)	C6	C5	N1
Cl1	Te1	Cl2[1]	90.49(5)	N1	C3	C4
Cl1[1]	Te1	Cl1	180.00(6)	C2	C1	N1
Cl1[1]	Te1	Cl3	90.26(4)			

Table S21. Bond Angles for o-$(Et_3NH)_2TeCl_6$ phase.

Atom	Atom	Atom	Angle/°	Atom	Atom	Angle/°
Cl0A	Te2	Cl12	91.67(8)	Cl4	Te1	Cl6
Cl10	Te2	Cl0A	89.68(8)	Cl4	Te1	Cl3
Cl10	Te2	Cl12	89.83(9)	Cl4	Te1	Cl1
Cl10	Te2	Cl9	178.28(8)	C17	N3	C13
Cl10	Te2	Cl11	89.77(8)	C17	N3	C15
Cl9	Te2	Cl0A	88.86(8)	C13	N3	C15
Cl9	Te2	Cl12	89.30(8)	N3	C17	C18

Cl8	Te2	Cl0A	88.23(9)	C14	C13	N3
Cl8	Te2	Cl12	179.54(9)	C16	C15	N3
Cl8	Te2	Cl10	90.63(9)	C3	N1	C5
Cl8	Te2	Cl9	90.24(9)	C3	N1	C1
Cl8	Te2	Cl11	88.11(9)	C1	N1	C5
Cl11	Te2	Cl0A	176.29(9)	C4	C3	N1
Cl11	Te2	Cl12	91.99(8)	C6	C5	N1
Cl11	Te2	Cl9	91.74(9)	N1	C1	C2
Cl2	Te1	Cl3	89.28(9)	C11	N2	C9
Cl6	Te1	Cl2	179.17(9)	C7	N2	C11
Cl6	Te1	Cl3	90.60(9)	C7	N2	C9
Cl6	Te1	Cl1	89.97(8)	N2	C11	C12
Cl5	Te1	Cl2	90.60(9)	C8	C7	N2
Cl5	Te1	Cl6	89.54(9)	C10	C9	N2
Cl5	Te1	Cl3	179.13(8)	C19	N4	C21
Cl5	Te1	Cl1	90.80(8)	C19	N4	C23
Cl5	Te1	Cl4	89.68(8)	C23	N4	C21
Cl1	Te1	Cl2	89.21(9)	N4	C19	C20
Cl1	Te1	Cl3	90.06(8)	C22	C21	N4
Cl4	Te1	Cl2	89.74(9)	N4	C23	C24

Table S22. Bond Angles for $(Et_3NH)_2Te_2Cl_{10}$ phase.

**Atom**	**Atom**	**Atom**	**Angle/°**	**Atom**	**Atom**	**Angle/°**
Cl3	Te1	Cl2	89.12(14)	Cl5	Te1	Cl3
Cl3	Te1	Cl2[1]	86.00(15)	Cl5	Te1	Cl4
Cl4	Te1	Cl3	179.63(15)	Cl5	Te1	Cl2[1]
Cl4	Te1	Cl2[1]	93.64(15)	Cl5	Te1	Cl2
Cl4	Te1	Cl2	90.77(14)	Te1	Cl2	Te1[1]
Cl2	Te1	Cl2[1]	87.16(11)	C5	N1	C2
Cl1	Te1	Cl3	90.3(2)	C5	N1	C4
Cl1	Te1	Cl4	90.0(2)	C2	N1	C4
Cl1	Te1	Cl2[1]	175.56(18)	N1	C5	C6
Cl1	Te1	Cl2	90.31(18)	C1	C2	N1
Cl1	Te1	Cl5	91.87(19)	C3	C4	N1

[1]1-X,1-Y,1-Z

Table S23. Characteristics of N-H···Cl hydrogen bonds in $(Et_2NH_2)_2TeCl_6$, *m*-, and *o*-$(Et_3NH)_2TeCl_6$ phases

N-H	d(N-H), Å	d(H···Cl), Å	< NHCl, deg.	d(N···Cl), Å	Cl
			**$(Et_2NH_2)_2TeCl_6$**		
N1-H1A	0.890	2.485	166.35	3.356	Cl1
N1-H1B	0.890	2.678	134.37	3.361	Cl2
N1-H1B	0.890	2.592	140.17	3.324	Cl3
			***m*-$(Et_3NH)_2TeCl_6$**		
N1-H1	0.980	2.694	137.57	3.481	Cl2
N1-H1	0.980	2.553	136.70	3.335	Cl3
			***o*-$(Et_3NH)_2TeCl_6$**		
N3-H3	0.980	2.255	166.56	3.216	Cl12
N1-H1	0.980	2.327	160.10	3.266	Cl0A
N2-H2	0.980	2.566	162.84	3.514	Cl12
N4-H4	0.980	2.664	167.97	3.628	Cl9

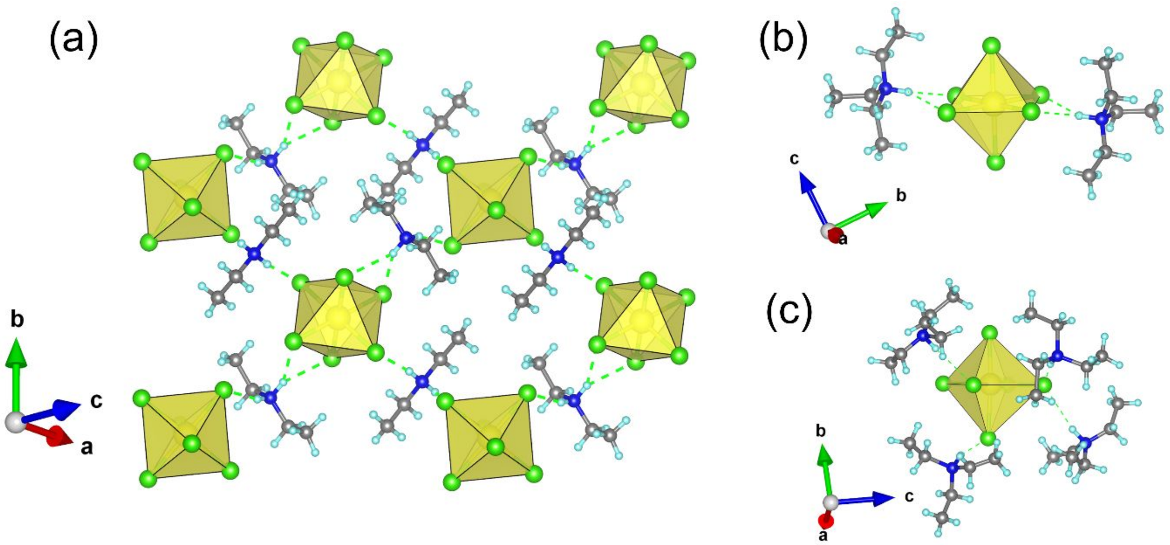

Figure S1. Different dimensionalities of clusters built on N-H···Cl hydrogen bonds in $(Et_2NH_2)_2TeCl_6$, α-, and β-$(Et_3NH)_2TeCl_6$ phases: (a) 2D layer in the $(Et_2NH_2)_2TeCl_6$ structure; (b) 0D $(Et_3NH)_2TeCl_6$ unit in α-$(Et_3NH)_2TeCl_6$ phase; (c) 0D $(Et_3NH)_4TeCl_6^{2+}$ unit in β-$(Et_3NH)_2TeCl_6$ phase. Hydrogen bonds are depicted as green dashed lines.

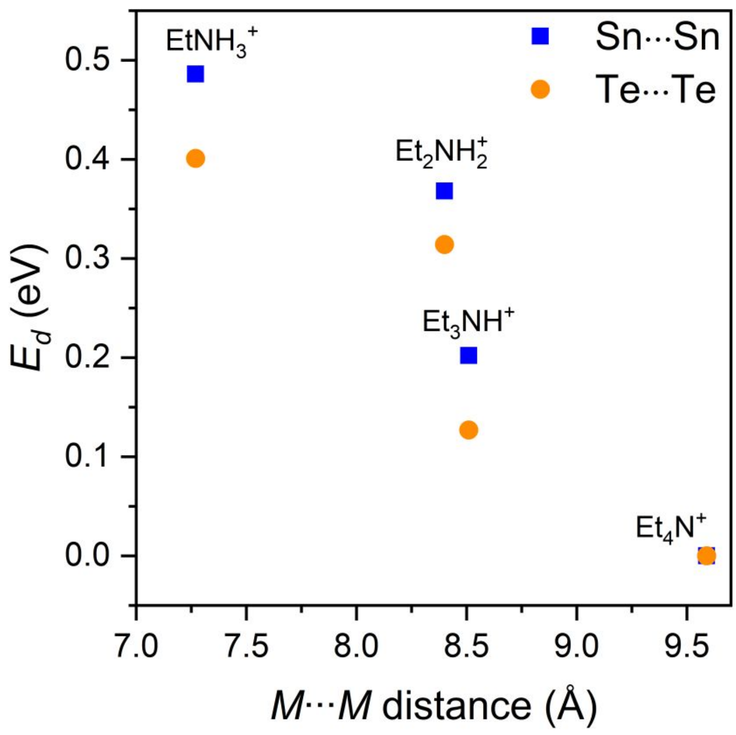

Figure S2. Bands dispersion in $(Et_nNH_{4-n})_2Sn_{1-x}Te_xCl_6$ samples

Table S24. EDS results for $(Et_nNH_{4-n})_2Sn_{1-x}Te_xCl_6$ samples (n = 3 or 4).

Sample	Loading composition, at. %			EDS, at. %		
	**Sn**	**Te**	**Cl**	**Sn**	**Te**	**Cl**
$(Et_3NH)_2Sn_{0.9}Te_{0.9}Cl_6$	12.86	1.43	85.71	17.30	1.70	81.00
$(Et_3NH)_2Sn_{0.7}Te_{0.3}Cl_6$	10.00	4.29	85.71	13.60	8.30	78.10
$(Et_3NH)_2Sn_{0.5}Te_{0.5}Cl_6$	7.14	7.14	85.71	9.70	13.00	77.30
$(Et_3NH)_2Sn_{0.3}Te_{0.7}Cl_6$	4.29	10.00	85.71	3.20	12.50	84.30
$(Et_4N)_2Sn_{0.9}Te_{0.9}Cl_6$	12.86	1.43	85.71	14.00	1.40	84.60
$(Et_4N)_2Sn_{0.7}Te_{0.3}Cl_6$	10.00	4.29	85.71	10.90	4.40	84.70
$(Et_4N)_2Sn_{0.5}Te_{0.5}Cl_6$	7.14	7.14	85.71	9.20	9.20	81.60
$(Et_4N)_2Sn_{0.3}Te_{0.7}Cl_6$	4.29	10.00	85.71	4.90	11.70	83.40

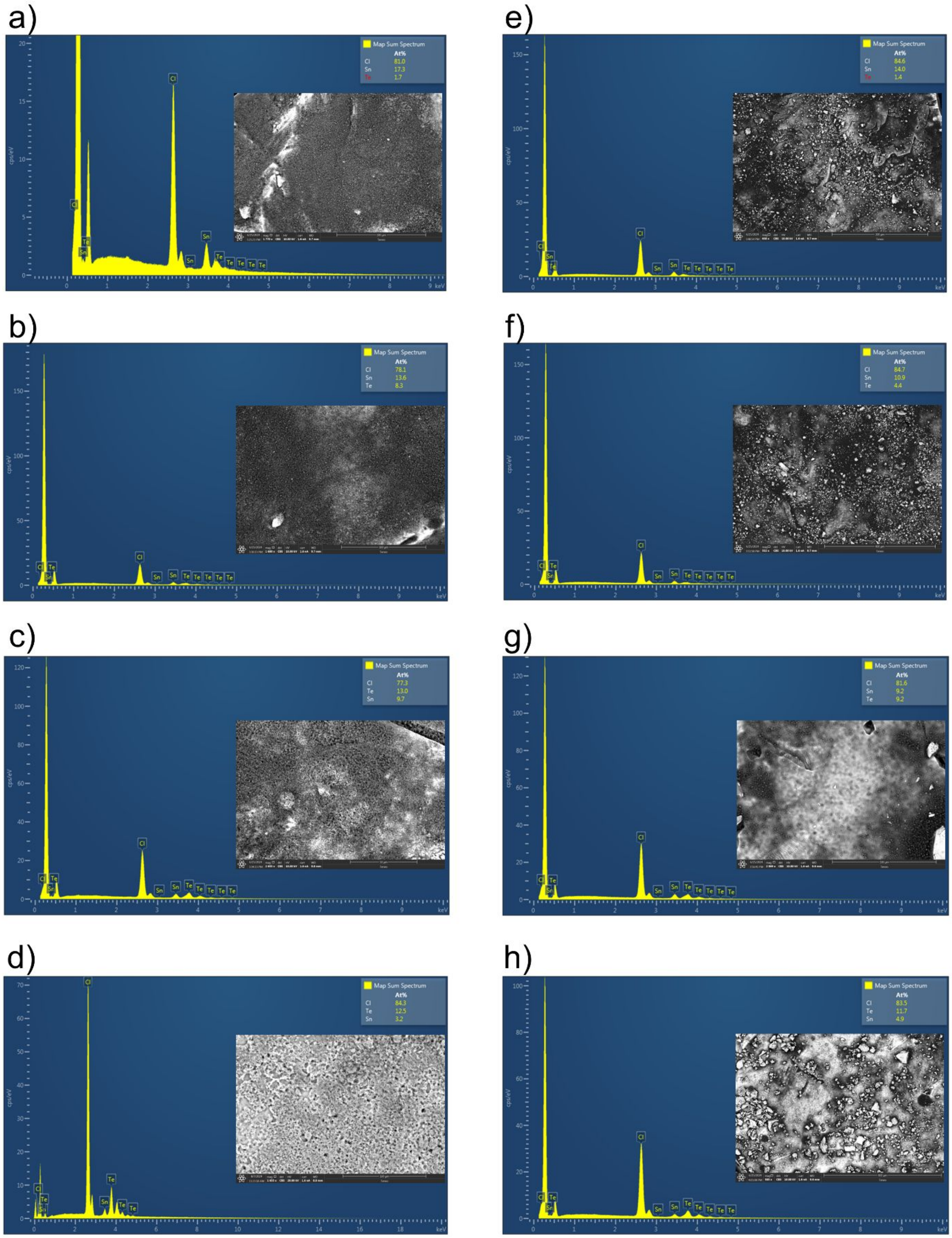

Figure S3. EDS results and SEM images for $(Et_3NH)_2Sn_{1-x}Te_xCl_6$ samples (x = 0.1 (a), 0.3 (b), 0.5 (c), 0.7 (d)) and $(Et_4N)_2Sn_{1-x}Te_xCl_6$ samples (x = 0.1 (e), 0.3 (f), 0.5 (g), 0.7 (h)).

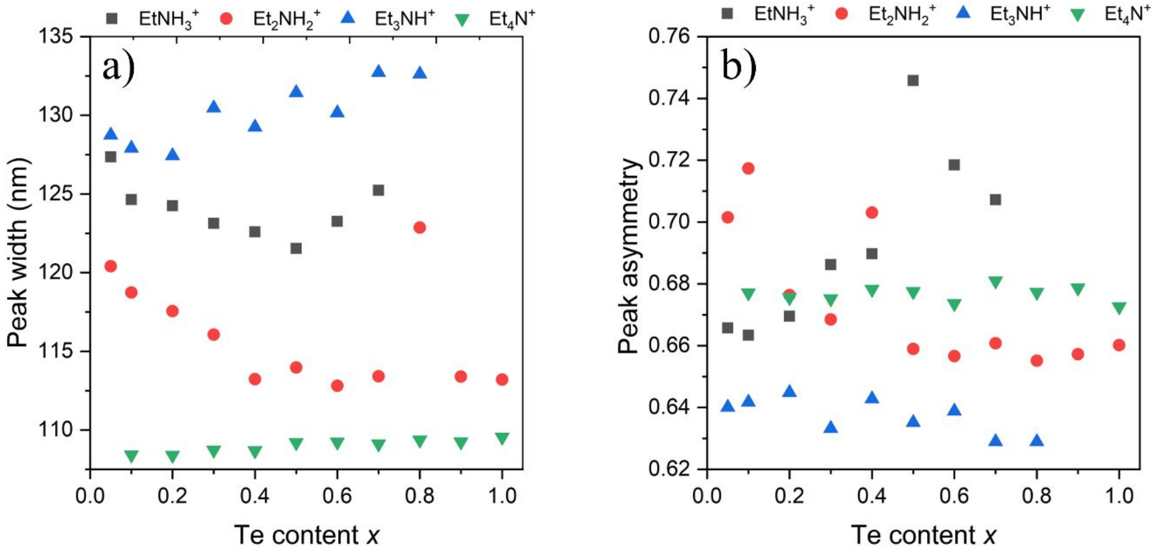

Figure S4. PL peak width (a) and asymmetry (b) in $(Et_nNH_{4-n})_2Sn_{1-x}Te_xCl_6$ samples. Bi-Gaussian function was used for peak fitting.

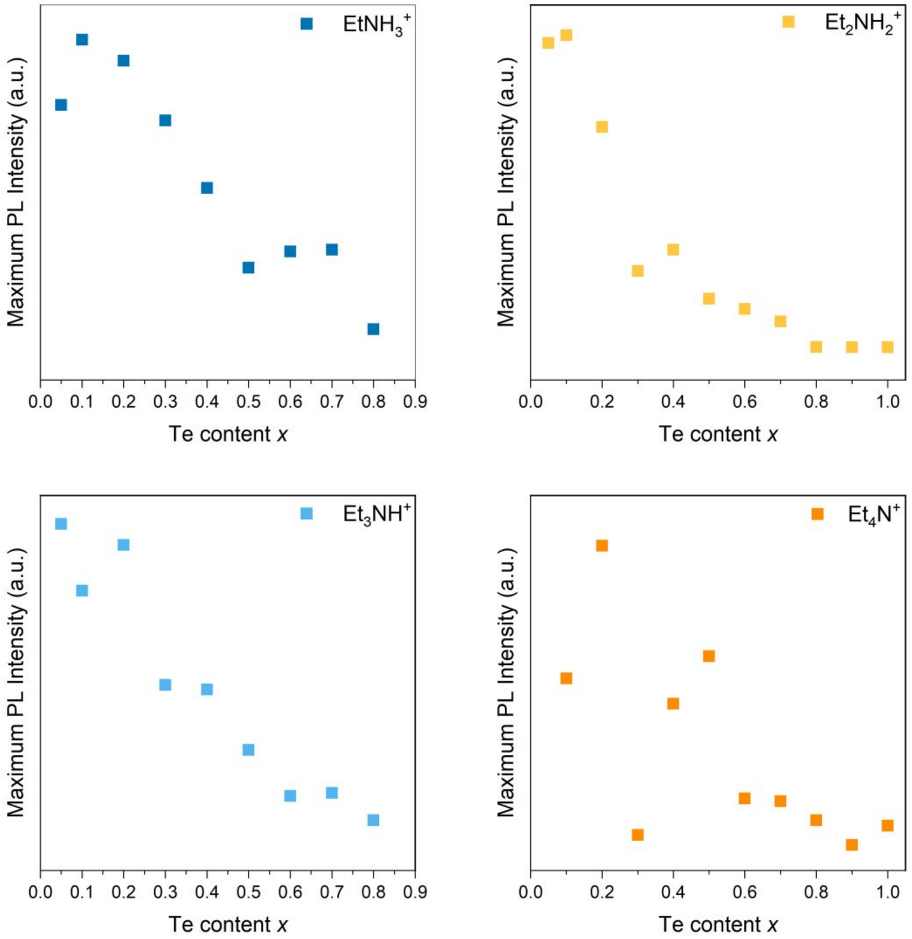

Figure S5. Maximum PL intensity in $(Et_nNH_{4-n})_2Sn_{1-x}Te_xCl_6$ samples

Table S25. Average PL lifetimes (ns) in $(Et_nNH_{4\text{-}n})_2Sn_{1\text{-}x}Te_xCl_6$ samples.

	n = 1		n = 2		n = 3		n = 4	
**x**	$\tau_{Av.Int.}$	$\tau_{Av.Amp}$	$\tau_{Av.Int.}$	$\tau_{Av.Amp}$	$\tau_{Av.Int.}$	$\tau_{Av.Amp}$	$\tau_{Av.Int.}$	$\tau_{Av.Amp}$
**1.0**	-	-	3.81(2)	3.15(5)	-	-	172(1)	155(1)
**0.9**	-	-	3.63(3)	3.46(1)	-	-	179(1)	163(2)
**0.8**	2.8(2)	0.78(2)	4.0(1)	3.58(3)	278(6)	245(3)	197(1)	177(2)
**0.7**	7.38(3)	5.56(5)	6.7(2)	4.42(3)	319(8)	258(3)	160(1)	145(2)
**0.6**	7.17(3)	4.63(4)	10.5(8)	5.7(2)	390(8)	287(3)	162(2)	146(2)
**0.5**	7.56(4)	5.24(5)	15.5(5)	8.1(2)	549(8)	370(4)	169(1)	159(2)
**0.4**	8.76(4)	7.29(8)	19(1)	10.9(5)	689(9)	470(5)	172(2)	160(3)
**0.3**	9.38(3)	9.38(3)	25.2(5)	15.81(9)	721(7)	512(5)	173(2)	155(2)
**0.2**	8.35(2)	7.60(4)	31.5(7)	23.8(3)	889(7)	691(6)	183(1)	169(2)
**0.1**	8.86(3)	8.86(3)	36.9(7)	31.1(2)	903(6)	784(7)	189(1)	175(2)
**0.05**	9.1(3)	8.8(3)	36(1)	32.9(2)	959(6)	885(8)	-	-

Table S26. PLQY in $(Et_nNH_{4\text{-}n})_2Sn_{1\text{-}x}Te_xCl_6$ samples.*

	n = 1	n = 2	n=3	n=4
**x**	PLQY, %			
**1.0**	Assumed to be < 1%		-	3.28
**0.9**	Assumed to be < 1%		-	3.44
**0.8**	Assumed to be < 1%		4.58	2.51
**0.7**	Assumed to be < 1%		4.53	2.88
**0.6**	Assumed to be < 1%		5.11	3.39
**0.5**	Assumed to be < 1%		6.28	3.04
**0.4**	Assumed to be < 1%		7.84	4.61
**0.3**	Assumed to be < 1%		9.78	3.64
**0.2**	Assumed to be < 1%		14.26	4.07
**0.1**	<1%	<1%	19.85	4.58
**0.05**	Assumed to be < 1%		20.05	-

* For n = 1 and 2 samples with x = 0.1 showed PLQY < 1%. The data were not collected for the rest of the series as concentration quenching in these two systems makes PLQY even lower at higher Te content based on the intensities in Figure 6.

Table S27. Parameters of $TeCl_6^{2-}$ octahedra distortion in $(Et_nNH_{4-n})_2TeCl_6$ (n=1-4) phases*

Phase	Atom	Δ_d	Σ, deg	D_a, Å	G_3
$(EtNH_3)_2TeCl_6$	Te1	0	48.24	0	0.08351
$(Et_2NH_2)_2TeCl_6$	Te1	2.9×10^{-5}	3.23	0	0.08334
m-$(Et_3NH)_2TeCl_6$	Te1	1.6×10^{-4}	9.88	0	0.08337
$(Et_4N)_2TeCl_6$	Te1	1.7×10^{-4}	9.14	0	0.08335

* $\Delta_d = \frac{1}{6}\sum_{i=1}^{6}\left(\frac{d_i - d_{mean}}{d_{mean}}\right)^2$;

$\Sigma = \sum_{i=1}^{12}|90 - \varphi_i|$;

For an ideal octahedron $G_3 = 0.08255$.[1–3]

Table S28. Synthesis of $(Et_nNH_{4-n})_2TeCl_6$ (*n*=1-3) and $(Et_3NH)_2Te_2Cl_{10}$ crystals and $(Et_nNH_{4-n})_2Sn_{1-x}Te_xCl_6$ phases

Crystals	Reagents	Conditions
$(EtNH_3)_2TeCl_6$	TeO_2, 0.14g, 0.88 mmol<br>Ethylamine 66-72 % water solution, ρ = 0.81 g/ml, 35 μl, 0.31 mmol	TeO$_2$ was dissolved in excess concentrated HCl (1.5-2 ml) on heating and stirring and transparent yellow solution formed. The corresponding amine was carefully added to this solution on stirring. Reactions yielded yellow crystals, which were filtered on vacuum filtration, dried, and used for further characterization.
$(Et_2NH_2)_2TeCl_6$	TeO_2, 0.05g, 0.31 mmol<br>Diethylamine 99 %, ρ = 0.707 g/ml, 65 μl, 0.63 mmol	
m-$(Et_3NH)_2TeCl_6$	TeO_2, 0.1g, 0.63 mmol<br>Triethylamine 99 %, ρ = 0.726 g/ml, 261 μl, 1.35 mmol	
o-$(Et_3NH)_2TeCl_6$	TeO_2, 0.1g, 0.63 mmol<br>Triethylamine 99 %, ρ = 0.726 g/ml, 166 μl, 1.26 mmol	
$(Et_3NH)_2Te_2Cl_{10}$	TeO_2, 0.1g, 0.63 mmol<br>Triethylamine 99 %, ρ = 0.726 g/ml, 87 μl, 1.19 mmol	

Series	Reaction	Conditions
$(EtNH_3)_2Sn_{1-x}Te_xCl_6$	$(1-x)SnO_2 + xTeO_2 + EtNH_2$	Reagents loaded in hydrothermal vessels with excess concentrated HCl (2-2.5 ml), heated to 190 °C and kept at this temperature for 3 h. Crystals formed upon hydrothermal reactions.
$(Et_2NH_2)_2Sn_{1-x}Te_xCl_6$	$(1-x)SnO_2 + xTeO_2 + Et_2NH$	Reagents loaded in hydrothermal

		vessels with excess concentrated HCl (2-2.5 ml), heated to 170 °C and kept at this temperature for 2 h. Crystals formed upon hydrothermal reactions.
$(Et_3NH)_2Sn_{1-x}Te_xCl_6$	$(1\text{-}x)SnO_2 + xTeO_2 + Et_3N$	Reagents loaded in hydrothermal vessels with excess concentrated HCl (1.5-2 ml), heated to 190 °C and kept at this temperature for 2.5 h. Crystals formed upon slow evaporation of clear solutions formed in hydrothermal reactions.
$(Et_4N)_2Sn_{1-x}Te_xCl_6$	$(1\text{-}x)SnO_2 + xTeO_2 + Et_4NHCl$	Reagents loaded in hydrothermal vessels with excess concentrated HCl (2 ml), heated to 190 °C and kept at this temperature for 2.5 h. Crystals formed upon hydrothermal reactions.

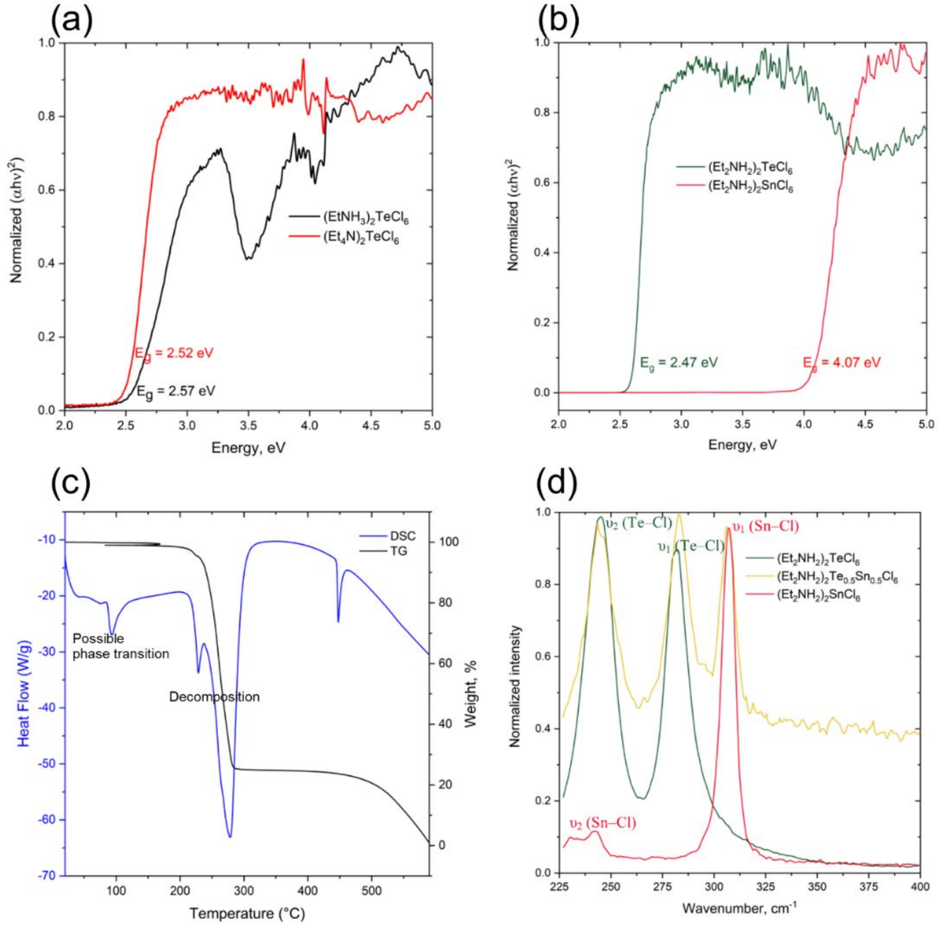

Figure S6. Tauc plots and optical band gaps for $(Et_2NH_2)_2TeCl_6$, $(Et_2NH_2)_2SnCl_6$, $(EtNH_3)_2TeCl_6$ and $(Et_4N)_2TeCl_6$ samples calculated from UV-vis reflectance data (a-b); DCS/TG

data for $(Et_2NH_2)_2TeCl_6$ sample (c); Raman spectra for $(Et_2NH_2)_2Sn_{1-x}Te_xCl_6$ (x = 0, 0.5, 1) samples (d).

References

(1) Lufaso, M. W.; Woodward, P. M. Jahn–Teller Distortions, Cation Ordering and Octahedral Tilting in Perovskites. *Acta Cryst B* **2004**, *60* (1), 10–20. https://doi.org/10.1107/S0108768103026661.

(2) McCusker, J. K.; Rheingold, A. L.; Hendrickson, D. N. Variable-Temperature Studies of Laser-Initiated 5T2 → 1A1 Intersystem Crossing in Spin-Crossover Complexes: Empirical Correlations between Activation Parameters and Ligand Structure in a Series of Polypyridyl Ferrous Complexes. *Inorg. Chem.* **1996**, *35* (7), 2100–2112. https://doi.org/10.1021/ic9507880.

(3) Blatov, V. A.; Serezhkin, V. N. Stereoatomic Model of the Structure of Inorganic and Coordination Compounds. *Russian Journal of Inorganic Chemistry* **2000**, *45* (Suppl. 2), S105–S222.
